# Father absence and gendered traits in sons and daughters

**DOI:** 10.1371/journal.pone.0179954

**Published:** 2017-07-05

**Authors:** Lynda G. Boothroyd, Catharine P. Cross

**Affiliations:** 1 Department of Psychology, Durham University, Durham, United Kingdom; 2 School of Psychology and Neuroscience, University of St Andrews, St Andrews, United Kingdom; University of Exeter, UNITED KINGDOM

## Abstract

Research has previously found a number of apparently contradictory patterns in the relationship between ‘father absence’ (having a non-resident father during childhood) and the expression of gender roles, as well as other sexually dimorphic traits such as aggression. In the current study we measured a battery of sexually differentiated traits in relation to family background. 133 men and 558 women from the United States and Australia completed the Bem Sex Role Inventory, the Barrett Impulsivity Scale, the Fear Survey Schedule and the Buss & Perry Aggression Questionnaire. Principal components analysis found two main axes of variation in these traits. Firstly, a general ‘reactivity’ factor, on which aggression, impulsivity, and fear all loaded positively, was weakly associated with father absence in women. Secondly, ‘masculinity’ (consisting of high scores on masculine traits, low fear, and physical and verbal aggression) was not associated with father absence. Participants (except American males) reporting a poor childhood relationship with their parents also had high ‘reactivity’ but not higher ‘masculinity’. We found some evidence of a link between father absence and earlier age of first coitus in American females (although not in Australia), but there was no link with age of menarche in either country. Overall, the current results suggest that previous findings linking gender development with father absence in girls may have arisen from a tendency towards greater externalising and reactive behaviour rather than a change in gender development per se.

## Introduction

Historically, interest in the developmental significance of ‘father absence’ (that is, non-residence of a child’s biological father for some or all of their childhood) has come from a number of sources. Father absence is currently primarily the focus of Evolutionary Anthropologists and Psychologists with an interest in predictors of life history variation and reproductive development (see e.g. [1, 2]). However, there also exist substantial Developmental Psychology literatures addressing father absence from the perspective both of psycho-social wellbeing and, more pertinently to the current paper, gender development (e.g. [3]). The literature on father absence and gender development tailed off following Stephenson & Black’s 1988 meta-analysis, with gender researchers growing interested in other developmental questions (for instance, the growing polarisation around constructivism ‘versus’ biological influences; see e.g. [4] for a review). More recent developments in the evolutionary literature, however, suggest that re-visiting the question of how father absence relates to sexually dimorphic behaviours may be warranted [5, 6]. In the current paper, we first take an overview of extant data regarding links between father absence, physiology and gender development. Second, we test the proposition that father absence may masculinise children’s developing sex roles and behaviour by comparing groups on multiple traits which are known to show sex differences in adults.

### Physical differences

#### Timing of puberty and reproductive behaviour

At least 16 studies in the last 25 years have found that father absence prior to puberty is associated with earlier menarche in daughters in Western samples (see e.g. [1] for a review) and there is evidence from studies of siblings that this association is environmental in nature [7]. Suggestions regarding the causation of this link have included the effects of stress on the endocrine system, the possible effects of exposure to familiar/stranger male pheromones and the effects of psychological reactions to stress on eating and therefore fat deposition (which is important in the timing of menarche). Because puberty is much harder to date retrospectively in men (lacking such a singular event as menarche), there has been far less research into father absence and sons’ development. Of what little exists, some has suggested that they too experience earlier puberty, while other researchers have found no links (see [8] for review) and others still have recently found father absence to predict *later* puberty in sons [9, 10].

In addition to timing of puberty, father absence and parent-child relationships are also associated with timing of first coitus and even first pregnancy in offspring (e.g. [11–13])–perhaps because of earlier physiological readiness for reproduction, or perhaps for more psychosocial reasons arising from (or related to) the parent-child relationship. One candidate psychological feature which might mediate a relationship between father absence and early coitus is impulsivity. Impulsivity has been linked not only with aggressive behaviour (as discussed below) but also with willingness to engage in casual sex with multiple partners [14].

#### Physical masculinisation

Waynforth [15] found that amongst the Maya of Belize, men with separated parents (who were all father absent) showed significantly greater craniofacial masculinity (as measured by the size of three key facial dimensions). Similarly, Boothroyd and Perrett [6] found amongst a British student sample, that parental separation (again, almost always resulting in the child becoming father absent) in early childhood was associated with increased ratings of facial masculinity in young women. Such differences could be of a genetic nature (i.e. androgenised parents have more likelihood of marital breakdown, and have more masculine offspring) although the evidence of environmental causation above regarding puberty may make this less likely. Alternatively the link here may also be environmental such that either: stress or perhaps pheromone exposure in childhood affects pubertal hormone levels and thus affects facial structure; or, perhaps less likely, masculinised offspring could in some way be a catalyst to marital breakdown. In either respect, these data suggested that father absence may be associated with physical androgenisation and as such, that broader patterns of behavioural differences between father absent and father present offspring, may also be explicable in these terms. A key challenge in addressing this question, however, is the fact that the vast majority of research has focused on one or two measures at a time and as such falls short of giving a holistic view of whether father absence is indeed a moderator of gendered behaviour. In this following section, therefore, we will consider the different aspects of gender researched in this context so far: explicit gender role, and aggression, fearfulness, and impulsivity, which all show sex differences by adulthood.

### Gender role

Research into father absence and gender role has historically produced rather mixed results. Some studies have found that father absence due to divorce was associated with low masculinity in boys (e.g. [16–18]) and others found no effect (e.g. [19]: our analyses of parental divorce/gender role frequencies presented in the paper gives χ^2^ = 2.87, *p* = .41). Stevenson & Black [3] attempted to synthesise the existing results in a meta-analysis of North American research up until 1986. They split studies on the basis of type of methodology, aspect of gender studied, age of subjects, and reason for father absence, amongst other factors. While there were insufficient studies to look at interactions between these factors, they did find that different groups of studies found different results. When split by participant age, confidence intervals confirmed that the significant association between father absence and feminine gender role was strongest in boys under 7 years of age, slight in 7–12 year olds and over 18s and absent in 13–17 year olds. This difference persisted in both lower and middle class samples (although not “unspecified” samples). More importantly, when they compared different aspects of gender, they found that amongst boys and men, father absence was associated with feminine gender orientation and preference, while it was associated with *masculine* gender adoption (I.e. sons of absent fathers seemed to think in a feminine way, but behave in a masculine way). This is concordant with Beatrice Whiting’s [20] suggestion that father-absent boys will have a feminine gender identity due to identification with the mother during childhood, and masculine behaviour as a reaction against this socially inappropriate feminine identity (‘protest masculinity’).

However, Stephenson & Black also split the studies on reasons for father absence. They found that father absence due to unspecified reasons (69 studies) or military service (7 studies) was associated with a more feminine gender role, but there were no overall effects of father’s death (7 studies) or parental divorce (33 studies). Whether this means that father absence due to divorce has no effect on gender, or whether in fact half of the ‘divorce’ studies show masculine *behaviour* while the other half show feminine *identity* as above, is impossible to say. Furthermore, they found no effects amongst studies of white boys, only amongst studies of Black or unspecified populations. It is therefore very unclear exactly how father absence, particularly father absence due to parental separation, relates to gender role development in males.

Similarly, there are mixed results in terms of the effects of father absence on girls’ gender development, although there have been far fewer studies looking at girls. Hetherington [21] found that teenage girls with divorced parents (who were all father absent), a dead father, or living with both married parents did not differ in terms of sex role. However, Vess et al [22] found that early parental divorce was associated with greater masculinity in girls, while late parental divorce (teens) was associated with femininity in girls (although the different groups were small and they assumed, but did not confirm, divorce to be synonymous with father absence). Stevenson & Black [3] found almost no overall effects of father absence on girls’ gender development. When the studies were split by participant’s age, 13–17 year old girls (10 studies) did show that father absence led to less sex-typed gender but there were no differences in any of the different groups for type of father absence, aspect of gender, race or socio-economic status (SES).

This apparent ambiguity in results is perhaps less surprising when one considers that the absence of the father from the family home does not necessarily imply the complete absence of the father from the child’s life. Furthermore, in all types of family, the relationship between the parents and the child can vary greatly. Later research into fathers and child’s gender therefore moved on to address the relationship with the father rather than his presence/absence per se. Unfortunately, however, this literature also found ambiguous results. For instance, Kelly & Worrell [23] and Jackson et al [24] both found that warm fathers were associated with androgynous sons (sons had high levels of both masculine and feminine traits on sex role tests), but while Jackson et al found the same pattern in daughters, Kelly & Worrell found no association between paternal warmth and sex role in daughters. Similarly, Orlofsky [25] found that rejecting fathers were associated with feminine sons and masculine daughters, but contrary to this, Stevenson [26] found that close fathers were also related to masculine daughters, and found no effect in sons. Most recently, Yang [27], looked at parent-child relationships and gender in Korean children and found no link between the two.

Other researchers have considered whether there are different socialisation patterns in different kinds of households. For instance, Biller [17] looked at mother’s encouragement of masculine/feminine behaviours and found that discouragement of masculine behaviours seemed to mediate the lack of masculine gender role in father-absent boys (see [28] for further discussion of the role of mother’s attitudes). On the other hand, some researchers have suggested that it is *fathers* which lead to greater gender typing in *intact* families. Siegal [29] conducted a meta-analysis of parental gender socialisation behaviours and found that although only half of 39 studies showed an effect of child gender on father’s behaviour, there was a significant trend across the studies for fathers to treat their children differently depending on their gender. The effect size for fathers was significantly greater than that for mothers, with the latter not differing significantly from zero.

Research in this area appears to have largely halted during the 1980s; a search of the literature (for publications citing Stevenson & Black and/or using the keywords: gender, masculinity, father absence, divorce) only revealed the work of Yang [27] as cited above and [18]. The latter found that father absence was associated with less traditional gender roles (i.e. less masculine sons and more masculine daughters) in African American teenagers, particularly amongst lower SES families. A body of recent research, however, has been conducted with children of lesbian couples. These studies have reported no differences in gender identity and adoption between children raised by lesbian couples (who do not have a father in the home) and children raised by heterosexual couples (see [30] for a review). This has two possible implications. Firstly it seems to support the notion that it is psychosocial stress and family attitudes which lead to differences (if any) between father-absent and father present individuals. Alternatively, it may suggest that some underlying genetic factor (such as androgenisation, as discussed below) mediates both parental relationship success and offspring gender identity–such that children whose mothers have more lasting adult relationships (be it a hetero- or homosexual relationship) may have different gender identity than children whose mothers experience relationship breakdown. More research is required, therefore, to determine exactly what about the absence of a father in the home causes differences in development.

### Specific sexually dimorphic behaviours

#### Aggression and delinquency

A key dimorphic trait of particular relevance to father absence is aggression. Physical aggression is a strongly sexually dimorphic trait, with differences appearing before 2 years of age [31] and continuing into adulthood. Male-male homicide vastly exceeds female-female homicide in all societies for which data exists (see e.g. [32, 33] for comprehensive reviews) and men consistently show higher levels of physical aggression than women across both laboratory studies and questionnaires (see e.g. [32, 34, 35]).

Differences between children from intact and father absent/separated households in rates of physical aggression were first identified (primarily amongst boys) in the mid-C20th (see e.g. [28] for discussion) and there is evidence that father absence has continued to be associated with aggression, delinquency and problem behaviours across later decades. For instance, Cherlin et al [36] found in a 1960s British sample and a 1970s American sample that parental divorce was associated with greater problem behaviours in children (although there was no difference in American girls). Hetherington [37] also found an association between father absence due to divorce (but not father’s death) and increased levels of aggression in teenage girls. Similarly, Berezckei & Czanaky [38] found using data from the late 1980s that boys whose parents were divorced and had been raised by their mothers (i.e. were father absent) engaged in significantly greater rule-breaking and delinquent behaviour during their teens than boys with intact families or with dead fathers. Again, however, they found no difference in girls. Stevenson & Black included aggression in their meta-analysis and found a significant overall association between father absence and aggression in both males (18 studies) and females (only 2 studies). Furthermore, there is cross cultural evidence that amongst patriarchal societies, ‘father absence’ (as indexed in the ethnographic record by a greater recorded sleeping distance between fathers and infants) is associated with higher levels of assault and homicide [39].

#### Fearfulness and impulsivity

Fearfulness and impulsivity have a relevance to the father absence literature which is more secondary. Fearfulness is strongly sexually dimorphic, with women showing higher levels of phobias than men, rating identical situations as more fear-provoking, and being less likely to engage in physically dangerous activities [40, 41]. Although men tend to show higher levels of social fears, women are more likely to be afraid of anything which could cause injury (e.g. animals, heights, injections: [40]). Similarly, there is a significant sex difference in impulsivity across development (e.g. [42, 43]).

The gender differences in fearfulness and behavioural impulsivity have both been suggested as the psychological mechanism underlying the gender differences in physical aggression (see e.g. [44] for a review). One could therefore predict that the elevated levels of physical aggression in children of divorcees ought to be associated with lower levels of fearfulness and greater behavioural impulsivity. However, an extensive search of the literature yielded only one study which had measured fearfulness in children from different family backgrounds. Milne et al [45] observed that children living with both biological parents had lower levels of phobias than children who did not live with both parents. However, whether these latter children were specifically the children of separated parents (who tend to be, but are not always, father absent), or whether they also included children who were adopted, fostered or living with step-parents (all of which may be associated with behavioural problems) was not clear.

Regarding impulsivity, parental separation is associated with developmental disorders characterized by impulsivity and poor inhibitory control, such as ADHD and conduct disorder [46, 47] and there may be a link between father absence and non-pathological impulsivity. In early research, Mischel [48] found a link between father absence and reduced delay in gratification amongst Trinidadian children, and Cortes and Fleming [49] found father absent boys were reported as more impulsive and irritable than father present boys using an economically deprived African American sample. Young and Parish [50] found no link between father absence (of any kind) and cognitive impulsivity in college age women, using the matching family figures test. More recently, however, Rostad and colleagues [51] reported that great father psychological presence/closeness is associated with less impulsivity in college women (although they do not report a relationship with parental separation). Apart from these studies, it is difficult to find evidence using psychometric or laboratory assessments of impulsivity; research using other behaviors has however, found a link between father absence and, gambling and drug use [52], and sexual risk taking (e.g. [2, 53], although cf [54]).

### Summary

Overall, there appear to be four contradictory aspects of the development of father-absent offspring compared to father-present offspring: (1) they appear to have no difference in, or feminisation of, their gender role identification; (2) they have no difference or masculinisation of their gender role adoption (particularly including elevated aggression and delinquency); (3) limited evidence suggests they have increased levels of sub-clinical fears (an apparently anti-masculine feature); (4) they may be more impulsive in some domains; (5) they have a masculinised appearance in at least 2 studies. These factors appear to defy theoretical integration. Thus far it appears no research has sought to study these traits simultaneously within the same population, which is essential if we are to try and understand how they link together.

The primary purpose of this paper was therefore to investigate the links between family structure during childhood and sexual dimorphism in multiple measures of behaviour and personality. Our hypothesis was that father absence and poorer quality family relationships are associated with greater general behavioural and psychological masculinity across both sexes. Participants were assessed using standardised self-report measures for aggression, impulsivity, fearfulness and sex role identity. These outcomes were then entered into a principal components analysis to investigate whether they would load onto a common ‘masculinity’ factor, such that a more masculine sex role would be associated with greater physical aggression, greater impulsivity, and reduced fearfulness.

The factors derived from the principal components analyses, and indices of reproductive development (in this case, timing of puberty and first coitus) were compared between participant family backgrounds. Should Boothroyd & Perrett’s [6] hypothesis be correct, father absence is associated with hormonal masculinisation which drives both pubertal/physical changes and broader behavioural changes. We would therefore predict that both males and females with absent fathers will show a shift towards the more male-typical end of the general masculinisation scale (i.e. more physically aggressive, more impulsive, less fearful and a more masculine sex role identity). However, should other previous suggestions (e.g. [20]) be correct, the higher levels of aggression in father-absent boys are a reaction to an initially feminised sex role identity, while father-absent girls have a similarly less gender-typical identity but no such later reaction against that identity. We would therefore predict that our sex role and behaviour measures would not share common variance and that men with absent fathers should show a more feminine sex role identity, coupled with higher levels of aggression, while women with absent fathers would show more general masculinity.

### Samples

We tested the above hypotheses using a sample of young adult participants in the US and Australia, recruited via online repositories of social psychology research projects. Australia and the US are relatively high-parity nations in terms of gender empowerment: Their Global Gender Gap Index scores are 0.74 and 0.75, respectively [55]. The GGI ranges from 0, which represents the greatest possible inequality, to 1, representing perfect equality. Scores range from 0.51 (Yemen) to 0.86 (Iceland) for the 142 countries assessed in 2014. Australia and the US also have Global Gender Inequality indices of 0.11 and 0.28 respectively [56]. A GII of 0 represents ‘perfect’ gender equality and 1 represents the highest possible levels of inequality. Of the 155 countries with a GII in 2014, the range is 0.02 (Slovenia) to 0.74 (Yemen). Furthermore, Australia and the US have similar profiles in terms of Hofstede’s national masculinity-femininity index (Australia: 62, USA: 62), and the factor structure and sex differences found for the Bem Sex Role Inventory (BSRI: a self-report measure of the extent to which the respondent considers themselves to possess the personality and behavioural traits deemed more desirable in each sex) have been reported to be similar in Australia to those found in US samples [57]. Kashima et al [58] examined cross-cultural variation in sex differences in self-construal across five cultures including the mainland US and Australia (The others were Hawaii, South Korea, and Japan). They performed multidimensional scaling on measures of seven traits including agency, assertiveness, and emotional relatedness to others. They reported striking similarities between the mainland US and Australian samples with regard to the pattern of sex differences.

Some of our dimensions of interest show significantly greater sex differences within the US than other areas of the West, including Australia (sex differences in childhood aggression [59]; cultural expressions of sex roles as indexed by advertisements [60]). However, some sex differences are larger in Australian samples (e.g. physical aggression [34]; fearfulness in children [61]). With regard to impulsivity, Cross et al [43] found consistent evidence that men score higher than women in US samples, but not in other Western countries (including Europe and Australia). Their meta-analysis, however, only found one study with Barrett Impulsivity Scale (BIS) data from Australia with a sample size of only 31 [62]. So, while the Australian estimate (showing a small sex difference in the female direction) differed from the US mean, there is currently insufficient evidence that the two countries differ significantly with regard to sex differences in impulsivity.

Perhaps most crucially, there is evidence that parenting may influence relevant traits such as aggression similarly in the US and Australia [63]. Furthermore, a meta-analysis examining differences in conduct problems between children from intact vs divorced or separated families found no difference between US samples and ‘other’ samples (a broad category of which some were Australian samples [64]).

## Method

691 American and Australian participants (448 Australian, 133 men) aged 17 to 29 (mean = 20.5) were recruited online via links on social psychology research sites. They were invited to take part in a study “investigating the links between childhood experiences and adult personality”. The majority of participants indicated their ethnicity as ‘white’, and the vast majority reported being undergraduate students. Demographic details of participants from each country are given in Table 1. Participants reported whether they thought their parents’ income when they were children had fallen into the top, upper middle, lower middle or bottom quartile (these were left undefined by specific financial boundaries); the majority reported upper middle (41.5%), or lower middle (39.9%), followed by bottom (12.4%) and top (6.1%).

**Table 1:** Descriptive statistics for sample characteristics and raw outcome measure scores. (BSRI = Bem Sex Role Inventory; AQ = Aggression Questionnaire; FSS = Fear Survey Schedule; BIS = Barratt Impulsivity Scale.) Ns represent the number of participants providing data for each item/subscale.

	Australia			United States		
	N	%		N	%	
% female	448	78.6		243	84.8	
% White	412	66.5		179	84.4	
% students	448	94.92.0		243	73.3	
% Father (ever) absent	426	24.9		219	50.7	
	N	Mean	s.d.	N	Mean	s.d.
Mean age	448	19.51	(2.26)	243	22.27	(3.40)
Age of menarche	339	12.76	(1.45)	197	12.42	(1.42)
Age of first coitus	249	16.71	(2.10)	194	16.44	(2.20)
BSRI masculinity	427	4.39	(0.86)	204	4.62	(0.85)
BSRI femininity	426	4.71	(0.78)	204	4.98	(0.71)
AQ (anger)	405	2.59	(0.79)	161	2.46	(0.88)
AQ (hostility)	403	2.91	(0.82)	160	2.72	(0.91)
AQ (physical)	406	2.40	(0.86)	160	2.28	(0.84)
AQ (verbal)	403	3.00	(0.83)	160	2.89	(0.88)
FSS: total	417	2.16	(0.67)	194	2.00	(0.57)
BIS: attention	421	2.44	(0.41)	197	2.35	(0.46)
BIS: motor	424	2.36	(0.50)	198	2.19	(0.48)
BIS: planning	423	2.41	(0.38)	198	2.27	(0.37)

Ethical approval for the research was granted by the Science Faculty Ethics Committee at Durham University. Participants gave consent via button click and details of online sources of support for those experiencing distress relating to divorce were given at the close of the study.

Participants completed a series of questionnaires online, firstly giving the demographic data above, and then reporting on measures of the following variables (see text in S1 Text for items):

### Father absence

Participants reported whether their parents had separated, who they lived with and at what age any separation occurred. Participants were classified as *father absent* if their parents had separated before they were 12 and they continued to live with their mothers, or if they had only ever lived with their mothers. Where parental separation (followed by living with the mother) occurred after they were 12, they were classified as *father absent after 12*. Those who lived with their parents until they left home were classed as *father present*. Age 12 was used in order to match the family relationship data below. Unfortunately, there were insufficient father-absent male participants to treat the two father-absent groups separately (final Ns = 11 after 12 years, 17 before 12 years) and so they were amalgamated for analyses with males. For women, we ran an analysis comparing all three categories, which was followed up with a secondary analysis which used the same two categories as were used for men. Furthermore, because there were only six father absent males in the US sample who reported age of first coitus and only three who responded to all behavioural questionnaires, all US males were excluded from father absence analyses. Anyone whose parents died, or who lived only with their fathers, or whose parents had joint custody were coded as missing. Furthermore, anyone reporting ‘other’ was also coded as missing.

### Family relationships

Participants rated the warmth with which they recalled each parent, and the quality of their biological parents’ relationship, before the participant reached 12 years, on a 1–9 Likert scale. This measure has previously been used by Boothroyd & Perrett [6, 65] and has been shown to relate to bodily masculinity, age of menarche and facial preferences. It also correlates with Adult Attachment classification (using the Hazen & Shaver questionnaire; see Boothroyd & Perrett [6]). All three variables were entered into a principal components analysis, which yielded a single component containing all three measures positively loaded (loadings ranged from .77 to .88) and explained 71% of the variance. Scores from this component were used as a measure of family relationship quality thereafter.

#### Aggression

Participants completed the Buss & Perry Aggression Questionnaire (AQ) [66], a measure which was developed to assess aggression in school and college age samples in the United States, and which exhibits significant sex differences on the physical aggression scale both within and outwith the US such that males score higher than females (e.g. [66, 67]). Participants rated the extent to which 29 statements were characteristic of them on a 5 point scale. Mean scores were calculated for each of the four subscales: verbal aggression (e.g. “I can’t help getting into arguments when people disagree with me”), physical aggression (“Given enough provocation, I may hit another person”), anger (e.g. “I sometimes feel like a powder keg ready to explode”) and hostility (e.g. “I am suspicious of overly friendly strangers”).

### Impulsivity

Participants completed the Barrett Impulsivity Scale (BIS; [68]). As discussed above, although the BIS does not always show a sex difference, men score significantly higher than women on the BIS in the United States [43], and as such it was a pertinent measure for use in the current study. Participants reported on a 4 point Likert scale from Never/Rarely to Almost always/Always the frequency in which they engaged in a range of behaviours. Mean scores were calculated for the three ‘second order’ subscales: inattentiveness (e.g. “I often have extraneous thoughts when thinking”), motor impulsivity (e.g. “I do things without thinking”) and (lack of) planning (e.g. “I solve problems by trial and error”).

#### Fearfulness

Fearfulness was assessed using the Fear Survey Schedule (FSS; [69]). The FSS was originally a clinical tool for identifying phobias, but has become a commonly used research tool in documenting both overall and specific fears in individuals. Participants reported on a 5 point scale from ‘not at all’ to ‘very much’, how much they were disturbed by a range of items such as loud voices, journeys by different modes of transport, tests, and being ignored. Mean scores were calculated for overall fearfulness (using all items), and for ‘blood’ fears (e.g. receiving injections, seeing blood) and ‘animal’ fears (e.g. worms, mice) which are higher in women than in men [40].

### Sex role identity

The Bem Sex Role Inventory (BSRI; [70]) was used to assess levels of masculine and feminine sex role identity. The BSRI was developed in the United States to document variation in behaviours typically ascribed to men (masculinity) and women (femininity). Individuals can vary in the degree to which they exhibit traits in each scale independently; however, generally men have tended to score higher than women on masculinity and vice versa for femininity. Although over time so-called ‘masculine traits’ have become less exclusively male in participant self-reports, there still remains a sex difference in femininity scores in the US ([71], see also [72]), and we would anticipate likewise a sex difference in overall masculinity/femininity ratio. Participants rated how much ‘like me’ 60 personality characteristics were in 7 point Likert scales. From these, separate scores were calculated for masculine and feminine traits.

### Reproductive development

Participants reported the age in years at which they had their first menstrual period (menarche; female participants only), and first had sexual intercourse (first coitus).

### Data processing

#### Adjusting for age

All variables were assessed to determine whether age had any effect on an individual’s scores. Because age is thought to relate nonlinearly to aggression in particular, peaking in the late teens and early twenties before declining again (e.g. [33]), variables were assessed using both linear and quadratic functions. No outcome measures were found to be significantly predicted by age (see text in S1 Text for details), so no variables are adjusted for age.

#### Principal components analysis on outcome measures

We hypothesised that all our gender-related psychometric measures would load onto a single factor. To assess this hypothesis, all outcome variables were entered into a principal components analysis with loadings below 0.3 suppressed. No rotation was used as we were specifically looking for a single factor. In fact the analysis produced two main principal components, as shown in Table 2. Factor 1 consisted of all aggression subscales, all impulsivity subscales, and fear. It also had a negative loading for BSRI femininity. We conceptualise this factor as general reactivity–i.e. the propensity of the individual to behave impulsively and react to situations negatively (with aggression or fear). Factor 2 consists of higher physical and verbal aggression, higher scores on BSRI masculinity, and low fear (i.e. fearfulness loaded negatively onto the factor); as such it can be conceptualised as general masculinity since it encompasses variables which are known to be higher in males than females. As well as loading positively on factor 1, planning and attention impulsivity loaded negatively on factor 2 –we return to this in the discussion. Factor scores were then examined for sexual dimorphism and country differences using linear models. For reactivity, there was a significant effect of country, such that Australians were more reactive than Americans (*β* = -0.36, SE = 0.11, *p* < .001, partial η^2^ = 0.03), but no significant sex difference (*β* = 0.09, SE = 0.13, *p* = .46), or interaction between sex and country (*β* = -0.22, SE = 0.27, *p* = .41). For masculinity, there was a significant sex difference such that men scored higher than women (*β* = -0.33, SE = 0.12, *p* = .007, partial η^2^ = 0.02), and an effect of country such that masculinity was higher in the US than in Australia (*β* = 0.34, SE = 0.10, *p* < .001, partial η^2^ = 0.03), but no interaction (*β* = 0.16, SE = 0.26, *p* = .52).

**Table 2:** Factor structures and sex differences in outcome variables.

	Factor 1 ‘reactivity’	Factor 2 ‘masculinity’
BSRI femininity	-.40	
BSRI masculinity		.72
AQ (anger)	.83	
AQ (hostility)	.72	
AQ (physical)	.78	.33
AQ (verbal)	.71	.44
FSS: total	.31	-.32
BIS: attention	.58	-.56
BIS: motor	.62	
BIS: planning	.44	-.56
Eigenvalue	3.48	1.70
Variance explained	34.8%	17.0%
Female mean (SD)	-0.02 (0.99)	-0.08 (0.94)
Male mean (SD)	0.04 (1.01)	0.28 (1.07)

## Results

### Father absence and factor scores

We ran linear models with father absence and country as predictors of factor scores, for men and women separately. When women were classified as father present and father *ever* absent as the men were (see Table 3), there was a weak but significant effect of father absence, such that those who had ever experienced father absence were more reactive than those who remained father present (*β* = 0.29, SE = 0.14, *p* = .037, partial η^2^ = 0.01). However, when father-absent women were coded as father absent before or after 12, neither of these groups had significantly higher reactivity than the father present group (Father absent before 12: *β* = 0.31, SE = 0.18, *p* = .08; Father absent after 12: *β* = 0.27, SE = 0.20, *p* = .17).

**Table 3:** Regression analyses showing associations between father absence and outcome measures for female participants. (Reference category for Country is Australia).

		*β*	*SE*	*p*	*partial eta squared*	*Adjusted R*^*2*^
Factor 1 Reactivity	Country	-0.50	0.15	.001	.05	.04
	Father absence				.01	
	before 12	0.31	0.18	.08		
	after 12	0.27	0.20	.17		
	Country x Father absence				<.01	
	before 12	-0.02	0.28	.95		
	after 12	-0.30	0.35	.40		
Factor 2 Masculinity	Country	0.45	0.14	.002	.02	.03
	Father absence				<.01	
	before 12	0.29	0.17	.08		
	after 12	-0.27	0.18	0.14		
	Country x Father absence				.01	
	before 12	-0.59	0.26	.03		
	after 12	-0.06	0.32	.84		
First coitus	Country	0.31	0.32	.34	<.01	.04
	Father absence				.02	
	before 12	0.28	0.45	.54		
	after 12	.08	0.51	.88		
	Country x Father absence				.03	
	before 12^a^	-1.78	0.60	.003		
	after 12	-0.29	0.77	.71		
Menarche	Country	-0.33	0.18	.08	.01	.01
	Father absence				<.01	
	before 12	0.10	0.25	.68		
	after 12	-0.37	0.28	.19		
	Country x Father absence				<.01	
	before 12	-0.35	0.35	.31		
	after 12	0.66	0.46	.15		

^a^ reflects a weak, positive association between father absence before age 12 and age at first coitus in US but not in Australia

When women were classified as father present and father *ever* absent as the men were, there was no significant effect of having ever experienced father absence on masculinity scores (*β* = 0.04, SE = 0.13, *p* = .77). When women were coded as either father absent before 12, father absent after 12, or never father absent, neither group had significantly higher masculinity than the father present group (Father absent before 12: *β* = 0.29, SE = 0.17, *p* = .08 Father absent after 12: *β* = -0.27, SE = 0.18, *p* = .14), but there was a significant interaction between country and father absence before 12 (*β* = -0.59, SE = 0.26, *p* = .03), which suggested that there might be a significant effect of father absence before 12 in one country but not the other. We therefore ran analyses separately for the US and Australia. In the US, the effect of father absence before 12 was clearly non-significant (*β* = -0.30, SE = 0.24, *p* = .21) and in the non-hypothesised direction (i.e. father-absent women were less masculine), whereas the effect in Australia of father absence before 12 was in the predicted direction (i.e. father-absent women were more masculine), although very small in size and with a two-tailed p-value of .06 (*β* = 0.29, SE = 0.16, *p* = .06, partial η^2^ = 0.02)

Australian men showed no significant effect of father absence for reactivity scores (*β* = 0.16, SE = 0.28, *p* = .57) or masculinity scores (*β* = 0.18, SE = 0.32, *p* = .58, see Table 4)

**Table 4:** Regression analyses showing associations between father absence and outcome measures for (Australian) male participants.

	*β*	*SE*	*p*	*partial eta squared*	*Adjusted R*^*2*^
Factor 1 Reactivity	0.16	0.28	.57	.01	<.01
Factor 2 Masculinity	0.18	0.32	.58	<.01	<.01
First coitus	0.53	0.67	.43	.01	<.01

### Ratings of parents and factor scores

Regression analyses were conducted separately with male and female participants, to assess the association between family relationship scores and both factor scores (see Table 5). Country (US vs Australia) and the interaction between country and family scores were added to these regression models. Overall, participant perceptions of parental marital quality were negatively related to reactivity scores, such that participants whose parents had a better quality relationship were less reactive; this was true for both male (*β* = -0.53, SE = 0.11, *p* < .001, partial η^2^ = 0.21) and female (*β* = -0.33, SE = 0.06, *p* < .001, partial η^2^ = 0.08) participants (see Table 5). There was no significant interaction between country and family relationship score for men (*β* = 0.26, SE = 0.28, *p* = .35) or women (*β* = 0.11, SE = 0.10, *p* = .35). Participant perceptions of parental marital quality did not significantly predict masculinity scores for men (*β* = 0.19, SE = 0.13, *p* = .15) or women (*β* = 0.05, SE = 0.06, *p* = .42).

**Table 5:** Regression analyses showing associations between family relationship scores and outcome measures for female and male participants. (Reference category for Country is Australia).

			*β*	*SE*	*p*	*partial eta squared*	*Adjusted R*^*2*^
Factor 1 Reactivity	Female	Country	-0.39	0.10	<.001	.04	.10
		Family relationship quality	-0.33	0.06	<.001	.08	
		Country x family relationship	0.11	0.10	.26	<.01	
	Male	Country	-0.50	0.25	.052	.03	.23
		Family relationship quality	-0.53	0.11	<.001	.21	
		Country x family relationship	0.26	0.28	.35	<.01	
Factor 2 Masculinity	Female	Country	-0.39	0.10	<.001	.04	.10
		Family relationship quality	0.05	0.06	.42	<.01	
		Country x family relationship	0.11	0.10	.26	<.01	
	Male	Country	0.59	0.30	.053	.03	.03
		Family relationship quality	0.19	0.13	.15	.01	
		Country x family relationship	-0.39	0.34	.25	.02	
First coitus	Female	Country	-0.13	0.23	.57	<.01	.03
		Family relationship quality	-0.04	0.17	.81	.02	
		Country x family relationship^a^	0.56	0.22	.01	.02	
	Male	Country	0.16	0.56	.77	<.01	.00
		Family relationship quality	0.29	0.32	.36	<.01	
		Country x family relationship	-0.28	0.57	.62	<.01	
Menarche	Female	Country	-0.27	0.13	.04	<.01	.01
		Family relationship quality	-0.11	0.08	.19	<.01	
		Country x family relationship	0.22	0.13	.08	<.01	

^a^ reflects a significant association between FRQ and age at first coitus in US but not Australia

### Reproductive outcomes

Father absence did not significantly predict age of first coitus in Australian men (*β* = 0.53, SE = 0.67, *p* = .43, see Table 4). Women who were father absent before age 12 experienced first coitus significantly earlier than father present women in the United States (*β* = -1.50, SE = 0.40, *p* = < .001, partial η^2^ = 0.10; father absence pre-12: 15.5 years, father absent after 12 16.8 years, father present 17.0 years). This effect was not found in Australia (*β* = 0.28, SE = 0.45, *p* = .53, father absence pre-12: 17.0 years, father absent after 12 16.8 years, father present 16.7 years), and the significant interaction term for country (*β* = -1.78, SE = 0.60, *p* = .003) indicates that father absence before age 12 affects first coitus differently in these two samples. There was no evidence of an effect of father absence before age 12 on age at menarche in women (*β* = 0.10, SE = 0.25, *p* = .68), nor a significant interaction between country and father absence (see Table 3).

Regression revealed no significant associations between family ratings and age of first coitus in either sex (men: *β* = 0.29, SE = 0.32, *p* = .36; women: *β* = -0.04, SE = 0.17, *p* = .81), or menarche in women (*β* = -0.11, SE = 0.08, *p* = .19), except for an interaction term between family relationship scores and country in predicting age of first coitus in women: positive ratings of family relationships were associated with a higher age of first coitus in American women (*β* = 0.52, SE = 0.12, *p* = < .001, partial η^2^ = 0.07) but not Australian women (*β* = -0.04, SE = 0.16, *p* = .81).

### Socioeconomic status

Participants who were father absent before 12 (*β* = 0.44, SE = 0.08, *p* < .001, partial η^2^ = 0.05), but not those who were father absent after 12 (*β* = 0.08, SE = 0.11, *p* = .44), reported a significantly lower parental income than those who remained father present. Reporting a positive family relationship was also associated with a higher self-reported income (*β* = 0.36, SE = 0.05, *p* < .001, partial η^2^ = 0.08) However, there were no significant associations between income and any of our outcome measures (see text in S1 Text) and further analyses controlling for income (see text in S1 Text) produced results that were the same as those reported above.

## Discussion

The aim of the present research was to examine the behavioural and developmental correlates of childhood father absence in two similar Western countries, and establish whether these correlates could be explained as a general masculinisation of behaviour. Our principal components analysis indicated that this was not the case: two factors emerged, one of which did indeed show sex differences (but which also showed substantial country differences) and which was categorised as ‘masculinity’. The other factor, however, was not dimorphic and instead appeared to reflect ‘general reactivity’ (aggressiveness, fearfulness and impulsivity). Although father absence before age 18 in women was (weakly) associated with an increase in scores on reactivity, there was no link between father absence and ‘masculinity’ scores. Father-absent men were neither more reactive nor more masculine than father-present men. In both sexes, poor self-reported family relationships predicted higher reactivity scores, but were not associated with masculinity.

We found no evidence that father absence was associated with changes in the gendered behaviour (i.e. our ‘masculinity’ factor) of female participants, which is concordant with the results of Stephenson & Black’s [3] meta-analysis. Instead, father absence may predispose women to greater sensitivity to, and negative reactivity towards, the social environment in general. As such the data regarding elevated aggression in father-absent women/girls in the studies analysed by Stevenson & Black may in fact not be an index of behavioural masculinity (as Stevenson & Black’s meta-analysis categorises it) but may instead be due to this greater reactivity. This also suggests that, for girls at least, the differing results regarding the association between father absence and gender development in previous studies may be mixed because the studies are accessing psychological constructs other than gender itself. Our results are, however, consistent with more recent suggestions that elevated stress reactivity may be an adaptive response to early experiences of highly stressful conditions (see e.g. [73–75]). The association between poor quality family relationships and reactivity is particularly consistent with this hypothesis.

In young adult men, our results showed no association between father absence and ‘masculinity’, indexed by sex role identity, low fearfulness, and high aggression. This is not consistent with the evidence for physical masculinisation in father-absent men and with Stevenson and Black’s conclusions that behavioural measures at least showed a clear bias towards masculinity in father-absent males, nor with those studies which suggested that father absence was associated with a reduction in masculinity.

A point of incongruity within our data was the difference between results using father absence as the predictor versus ratings of family relationship quality. While differences in outcomes between father absent groups were weak, correlations with ratings of family quality were much more robust and, as mentioned above, the results for reactivity were consistent across the sexes. This would suggest either that family conflict results in changes in emotional development (i.e. an increase in reactivity), or that individuals who are more reactive in their late teens and twenties are more likely to view memories of their parents negatively. The fact that stronger results have been found using the subjective predictor variable (including the only link between reactivity and family background in men) than from the objective predictor variable would seem to suggest that recall biases might be influencing results. Alternatively, this analysis might suggests that poor quality parental relationships cause changes in development independent of any effects of father absence.

We found effects of childhood experiences on reproductive outcomes in our American participants that did not replicate in Australia. In American females, father absence before age 12 and negative ratings of family relationships were associated with age of first coitus. To our knowledge, this study includes the first evidence that family background variables may relate differently to behavioural and reproductive outcomes in young adulthood in these two countries. Caution should be taken with interpreting these interactions, however, particularly in light of the modest size of the American sample and the fact that there were too few father-absent American males for analysis. Furthermore, our preliminary data processing suggested that Australian participants scored higher on general reactivity than American participants, and lower on behavioural masculinity. It is possible that higher ‘baseline’ reactivity and masculinity in the US is what causes father absence to have an effect on reproductive outcome here but not in Australia. Future work should confirm the extent to which these differences are not an artefact of self-report.

A final point of note is that the relationship between aggression and fearfulness seems in the current sample to operate on two different bases–greater levels of physical aggression are indeed associated with lower levels of fear insofar as they both load on our factor of behavioural/psychological masculinity; this relationship may well be explained by underlying androgenisation and is concordant with the view of aggression posited by Campbell [35]. However, all forms of aggressiveness simultaneously load on our reactivity factor such that those who have more fears are also more hostile, angry and verbally and physically aggressive; these same individuals also seem to be more behaviourally impulsive. These two simultaneous relationships between aggression and fearfulness amongst a sample of young adults is consistent with conceptualisations of aggression as having different types: *instrumental* aggression is negatively related to fear-related factors such as harm avoidance, is not characterised by particularly high levels of emotional arousal, and is more typical of men than of women; while *expressive* aggression is positively related to fear, associated with high levels of emotional arousal, and is not consistently higher in men than in women [76]. Considering types of aggression may explain the sometimes differing findings regarding the relationship between aggression and fear, and between aggression and early life experience.

Important caveats in the current study include the fact that most of our participants were students (which may explain for instance why we found no effects of SES on outcome measures), and our reliance on self-report data. This is particularly pertinent considering our measure of fearfulness: while it is a standard measure of fears in self-report tests, it was not intended as a measure of trait fearfulness. New measures such as the Situated Fear Questionnaire [77] may prove useful for this type of work in the future. Related to this, although the Barrett Impulsivity Scale is one of the primary self-report methods for assessing impulsivity, it does not show reliable sex differences in all samples (see e.g. [43] for meta-analysis) and may thus have failed to tap into the precise construct we were aiming to test (while still being clearly relevant to family background as our results show). Finally, our family data were entirely retrospective child-reports; longitudinal, third-party assessments of family relationships would allow for a better test of whether the marital relationship itself is critical in the development of these outcomes, or father absence specifically.

A final caveat concerns the age cut-offs we used. While considerable attention has been given to the ‘effects’ of father absence prior to 5–7 years, we selected a cut-off of age 12 for these family recall questions in order to give a realistically accessible window of memory for participants (i.e. the time roughly before they started high school). We likewise used 12 as a cut-off for our father absence analyses in women to be concordant with that. Unsurprisingly, women who were father absent between the ages of 12 and 18 do not appear to differ in reproductive outcomes from women who were never father absent–father absence having probably occurred too late to influence the onset of menarche, for example. However, when we compared ever father absent with never father absent groups (initially a response to having insufficient data from men for a finer-grained analysis), we found effects of this variable on reactivity in adulthood. This suggests that father absence might have effects on offspring more generally even when it occurs too late to influence pubertal development. Alternatively, people who are generally reactive might be more likely to have parents who separate for other reasons.

Our study revealed a relatively consistent picture of the effects of father absence in young women: women who were ever father absent showed slightly higher reactivity—but not higher masculinity—than women who remained father present until leaving home. This effect was not moderated by country. Women who were father absent before 12 also experienced earlier first coitus than father present women, but only in the US. A poorer quality of parental relationship, as assessed by subjective recall, was also associated with elevated reactivity in women, irrespective of country. These results suggest that father absence–and, more broadly, a stressful childhood or adolescence–result in a general increase in reactive behaviour in women, but not with ‘masculinisation’ of behaviour. For men the picture was less clear, partly because we did not have enough father absent men from the US for analysis. In Australia, however, father absence was not associated with increased ‘masculinity’ scores, increased reactivity, or earlier first coitus. Poor parental relationship quality was associated with increased reactivity in men, however, as it was in women. While our results illustrate the need for further cross-cultural research on the effects of father absence with large, representative samples–particularly of men—our results from women suggest that previous mixed reports of father absence in girls causing greater ‘masculinity’ might be attributable to greater reactivity being construed as masculinisation.

## Supporting information

S1 Text Questionnaire items and further analysis of age and income variables. (DOCX)

S2 Text Analysis code (RMarkdown). (RMD)

S3 Text Raw data file. (TXT)

S4 Text Notes on data file. (TXT)
