# Supplementary material for: Father absence and gendered traits in sons and daughters
Source: PLoS One. 2017 Jul 5;12(7):e0179954. doi: 10.1371/journal.pone.0179954 (PMC5497959; doi:10.1371/journal.pone.0179954)
Supplement: S1 Text — (DOCX) [file pone.0179954.s001.docx]

# S1.1 Questionnaire items

Your Age:

Your sex:

**Aggression Questionnaire**

Instructions:

Using the 5 point scale shown below, indicate how uncharacteristic or characteristic each of the following statements is in describing you. Place your rating in the box to the right of the statement.

1 = extremely uncharacteristic of me

2 = somewhat uncharacteristic of me

3 = neither uncharacteristic nor characteristic of me

4 = somewhat characteristic of me

5 = extremely characteristic of me

| 1. | Some of my friends think I am a hothead |  |
| --- | --- | --- |
| 2. | If I have to resort to violence to protect my rights, I will. |  |
| 3. | When people are especially nice to me, I wonder what they want. |  |
| 4. | I tell my friends openly when I disagree with them. |  |
| 5. | I have become so mad that I have broken things. |  |
| 6. | I can’t help getting into arguments when people disagree with me. |  |
| 7. | I wonder why sometimes I feel so bitter about things. |  |
| 8. | Once in a while, I can’t control the urge to strike another person. |  |
| 9. | I am an even-tempered person. |  |
| 10. | I am suspicious of overly friendly strangers. |  |
| 11. | I have threatened people I know. |  |
| 12. | I flare up quickly but get over it quickly. |  |
| 13. | Given enough provocation, I may hit another person. |  |
| 14. | When people annoy me, I may tell them what I think of them. |  |
| 15. | I am sometimes eaten up with jealousy. |  |
| 16. | I can think of no good reason for ever hitting a person. |  |
| 17. | At times I feel I have gotten a raw deal out of life. |  |
| 18. | I have trouble controlling my temper. |  |
| 19. | When frustrated, I let my irritation show. |  |
| 20. | I sometimes feel that people are laughing at me behind my back. |  |
| 21. | I often find myself disagreeing with people. |  |
| 22. | If somebody hits me, I hit back. |  |
| 23. | I sometimes feel like a powder keg ready to explode. |  |
| 24. | Other people always seem to get the breaks. |  |
| 25. | There are people who pushed me so far that we came to blows. |  |
| 26. | I know that “friends” talk about me behind my back. |  |
| 27. | My friends say that I’m somewhat argumentative. |  |
| 28. | Sometimes I fly off the handle for no good reason. |  |
| 29. | I get into fights a little more than the average person. |  |

**Fear Survey Schedule**

How scared would each of these things make you?

|  | \| Not at all \| A bit \| A fair amount \| Much \| Very much \| \| --- \| --- \| --- \| --- \| --- \| |
| --- | --- | --- | --- | --- | --- | --- |
| 1. Noise of vacuum cleaners | \| 1 \| 2 \| 3 \| 4 \| 5 \| \| --- \| --- \| --- \| --- \| --- \| |
| 2. Open Wounds | \| 1 \| 2 \| 3 \| 4 \| 5 \| \| --- \| --- \| --- \| --- \| --- \| |
| 3. Being alone | \| 1 \| 2 \| 3 \| 4 \| 5 \| \| --- \| --- \| --- \| --- \| --- \| |
| 4. Being in a strange place | \| 1 \| 2 \| 3 \| 4 \| 5 \| \| --- \| --- \| --- \| --- \| --- \| |
| 5. Loud voices | \| 1 \| 2 \| 3 \| 4 \| 5 \| \| --- \| --- \| --- \| --- \| --- \| |
| 6. Dead people | \| 1 \| 2 \| 3 \| 4 \| 5 \| \| --- \| --- \| --- \| --- \| --- \| |
| 7. Speaking in public | \| 1 \| 2 \| 3 \| 4 \| 5 \| \| --- \| --- \| --- \| --- \| --- \| |
| 8. Crossing streets | \| 1 \| 2 \| 3 \| 4 \| 5 \| \| --- \| --- \| --- \| --- \| --- \| |
| 9. People who seem insane | \| 1 \| 2 \| 3 \| 4 \| 5 \| \| --- \| --- \| --- \| --- \| --- \| |
| 10. Falling | \| 1 \| 2 \| 3 \| 4 \| 5 \| \| --- \| --- \| --- \| --- \| --- \| |
| 11. Automobiles | \| 1 \| 2 \| 3 \| 4 \| 5 \| \| --- \| --- \| --- \| --- \| --- \| |
| 12. Being teased | \| 1 \| 2 \| 3 \| 4 \| 5 \| \| --- \| --- \| --- \| --- \| --- \| |
| 13. Dentists | \| 1 \| 2 \| 3 \| 4 \| 5 \| \| --- \| --- \| --- \| --- \| --- \| |
| 14. Thunder | \| 1 \| 2 \| 3 \| 4 \| 5 \| \| --- \| --- \| --- \| --- \| --- \| |
| 15. Sirens | \| 1 \| 2 \| 3 \| 4 \| 5 \| \| --- \| --- \| --- \| --- \| --- \| |
| 16. Failure | \| 1 \| 2 \| 3 \| 4 \| 5 \| \| --- \| --- \| --- \| --- \| --- \| |
| 17. Entering a room where other people are already seated | \| 1 \| 2 \| 3 \| 4 \| 5 \| \| --- \| --- \| --- \| --- \| --- \| |
| 18. High places on land | \| 1 \| 2 \| 3 \| 4 \| 5 \| \| --- \| --- \| --- \| --- \| --- \| |
| 19. Looking down from high buildings | \| 1 \| 2 \| 3 \| 4 \| 5 \| \| --- \| --- \| --- \| --- \| --- \| |
| 20. Worms | \| 1 \| 2 \| 3 \| 4 \| 5 \| \| --- \| --- \| --- \| --- \| --- \| |
| 21. Imaginary creatures | \| 1 \| 2 \| 3 \| 4 \| 5 \| \| --- \| --- \| --- \| --- \| --- \| |
| 22. Strangers | \| 1 \| 2 \| 3 \| 4 \| 5 \| \| --- \| --- \| --- \| --- \| --- \| |
| 23. Receiving injection | \| 1 \| 2 \| 3 \| 4 \| 5 \| \| --- \| --- \| --- \| --- \| --- \| |
| 24. Bats | \| 1 \| 2 \| 3 \| 4 \| 5 \| \| --- \| --- \| --- \| --- \| --- \| |
| 25. Journeys by train | \| 1 \| 2 \| 3 \| 4 \| 5 \| \| --- \| --- \| --- \| --- \| --- \| |
| 26. Journeys by bus | \| 1 \| 2 \| 3 \| 4 \| 5 \| \| --- \| --- \| --- \| --- \| --- \| |
| 27. Journeys by car | \| 1 \| 2 \| 3 \| 4 \| 5 \| \| --- \| --- \| --- \| --- \| --- \| |
| 28. Feeling angry | \| 1 \| 2 \| 3 \| 4 \| 5 \| \| --- \| --- \| --- \| --- \| --- \| |
| 29. People in authority | \| 1 \| 2 \| 3 \| 4 \| 5 \| \| --- \| --- \| --- \| --- \| --- \| |
| 30. Flying insects | \| 1 \| 2 \| 3 \| 4 \| 5 \| \| --- \| --- \| --- \| --- \| --- \| |
| 31. Seeing other people injected | \| 1 \| 2 \| 3 \| 4 \| 5 \| \| --- \| --- \| --- \| --- \| --- \| |
| 32. Sudden noises | \| 1 \| 2 \| 3 \| 4 \| 5 \| \| --- \| --- \| --- \| --- \| --- \| |
| 33. Dull weather | \| 1 \| 2 \| 3 \| 4 \| 5 \| \| --- \| --- \| --- \| --- \| --- \| |
| 34. Crowds | \| 1 \| 2 \| 3 \| 4 \| 5 \| \| --- \| --- \| --- \| --- \| --- \| |
| 35. Large open spaces | \| 1 \| 2 \| 3 \| 4 \| 5 \| \| --- \| --- \| --- \| --- \| --- \| |
| 36. Cats | \| 1 \| 2 \| 3 \| 4 \| 5 \| \| --- \| --- \| --- \| --- \| --- \| |
| 37. One person bullying another | \| 1 \| 2 \| 3 \| 4 \| 5 \| \| --- \| --- \| --- \| --- \| --- \| |
| 38. Tough looking people | \| 1 \| 2 \| 3 \| 4 \| 5 \| \| --- \| --- \| --- \| --- \| --- \| |
| 39. Birds | \| 1 \| 2 \| 3 \| 4 \| 5 \| \| --- \| --- \| --- \| --- \| --- \| |
| 40. Sight of deep water | \| 1 \| 2 \| 3 \| 4 \| 5 \| \| --- \| --- \| --- \| --- \| --- \| |
| 41. Being watched working | \| 1 \| 2 \| 3 \| 4 \| 5 \| \| --- \| --- \| --- \| --- \| --- \| |
| 42. Dead animals | \| 1 \| 2 \| 3 \| 4 \| 5 \| \| --- \| --- \| --- \| --- \| --- \| |
| 43. Weapons | \| 1 \| 2 \| 3 \| 4 \| 5 \| \| --- \| --- \| --- \| --- \| --- \| |
| 44. Dirt | \| 1 \| 2 \| 3 \| 4 \| 5 \| \| --- \| --- \| --- \| --- \| --- \| |
|  |  |
|  | \| Not at all \| A bit \| A fair amount \| Much \| Very much \| \| --- \| --- \| --- \| --- \| --- \| |
| 45. Crawling insects | \| 1 \| 2 \| 3 \| 4 \| 5 \| \| --- \| --- \| --- \| --- \| --- \| |
| 46. Sight of fighting | \| 1 \| 2 \| 3 \| 4 \| 5 \| \| --- \| --- \| --- \| --- \| --- \| |
| 47. Ugly people | \| 1 \| 2 \| 3 \| 4 \| 5 \| \| --- \| --- \| --- \| --- \| --- \| |
| 48. Fire | \| 1 \| 2 \| 3 \| 4 \| 5 \| \| --- \| --- \| --- \| --- \| --- \| |
| 49. Sick people | \| 1 \| 2 \| 3 \| 4 \| 5 \| \| --- \| --- \| --- \| --- \| --- \| |
| 50. Dogs | \| 1 \| 2 \| 3 \| 4 \| 5 \| \| --- \| --- \| --- \| --- \| --- \| |
| 51. Being criticized | \| 1 \| 2 \| 3 \| 4 \| 5 \| \| --- \| --- \| --- \| --- \| --- \| |
| 52. Strange shapes | \| 1 \| 2 \| 3 \| 4 \| 5 \| \| --- \| --- \| --- \| --- \| --- \| |
| 53. Being in an elevator | \| 1 \| 2 \| 3 \| 4 \| 5 \| \| --- \| --- \| --- \| --- \| --- \| |
| 54. Witnessing surgical operations | \| 1 \| 2 \| 3 \| 4 \| 5 \| \| --- \| --- \| --- \| --- \| --- \| |
| 55. Angry people | \| 1 \| 2 \| 3 \| 4 \| 5 \| \| --- \| --- \| --- \| --- \| --- \| |
| 56. Mice | \| 1 \| 2 \| 3 \| 4 \| 5 \| \| --- \| --- \| --- \| --- \| --- \| |
| 57. Blood | \|  \|  \|  \|  \|  \| \| --- \| --- \| --- \| --- \| --- \| |
| A -- Human | \| 1 \| 2 \| 3 \| 4 \| 5 \| \| --- \| --- \| --- \| --- \| --- \| |
| B -- Animal | \| 1 \| 2 \| 3 \| 4 \| 5 \| \| --- \| --- \| --- \| --- \| --- \| |
| 58. Parting from friends | \| 1 \| 2 \| 3 \| 4 \| 5 \| \| --- \| --- \| --- \| --- \| --- \| |
| 59. Enclosed places | \| 1 \| 2 \| 3 \| 4 \| 5 \| \| --- \| --- \| --- \| --- \| --- \| |
| 60. Prospect of a surgical operation | \| 1 \| 2 \| 3 \| 4 \| 5 \| \| --- \| --- \| --- \| --- \| --- \| |
| 61. Feeling rejected by others | \| 1 \| 2 \| 3 \| 4 \| 5 \| \| --- \| --- \| --- \| --- \| --- \| |
| 62. Airplanes | \| 1 \| 2 \| 3 \| 4 \| 5 \| \| --- \| --- \| --- \| --- \| --- \| |
| 63. Medical odors | \| 1 \| 2 \| 3 \| 4 \| 5 \| \| --- \| --- \| --- \| --- \| --- \| |
| 64. Feeling disapproved of | \| 1 \| 2 \| 3 \| 4 \| 5 \| \| --- \| --- \| --- \| --- \| --- \| |
| 65. Harmless snakes | \| 1 \| 2 \| 3 \| 4 \| 5 \| \| --- \| --- \| --- \| --- \| --- \| |
| 66. Cemeteries | \| 1 \| 2 \| 3 \| 4 \| 5 \| \| --- \| --- \| --- \| --- \| --- \| |
| 67. Being ignored | \| 1 \| 2 \| 3 \| 4 \| 5 \| \| --- \| --- \| --- \| --- \| --- \| |
| 68. Darkness | \| 1 \| 2 \| 3 \| 4 \| 5 \| \| --- \| --- \| --- \| --- \| --- \| |
| 69. Premature heart beats (missing a beat) | \| 1 \| 2 \| 3 \| 4 \| 5 \| \| --- \| --- \| --- \| --- \| --- \| |
| 70. Naked people | \|  \|  \|  \|  \|  \| \| --- \| --- \| --- \| --- \| --- \| |
| A-Nude men | \| 1 \| 2 \| 3 \| 4 \| 5 \| \| --- \| --- \| --- \| --- \| --- \| |
| B-Nude women | \| 1 \| 2 \| 3 \| 4 \| 5 \| \| --- \| --- \| --- \| --- \| --- \| |
| 71. Lightning | \| 1 \| 2 \| 3 \| 4 \| 5 \| \| --- \| --- \| --- \| --- \| --- \| |
| 72. Doctors | \| 1 \| 2 \| 3 \| 4 \| 5 \| \| --- \| --- \| --- \| --- \| --- \| |
| 73. People with deformities | \| 1 \| 2 \| 3 \| 4 \| 5 \| \| --- \| --- \| --- \| --- \| --- \| |
| 74. Making mistakes | \| 1 \| 2 \| 3 \| 4 \| 5 \| \| --- \| --- \| --- \| --- \| --- \| |
| 75. Looking foolish | \| 1 \| 2 \| 3 \| 4 \| 5 \| \| --- \| --- \| --- \| --- \| --- \| |
| 76. Losing control | \| 1 \| 2 \| 3 \| 4 \| 5 \| \| --- \| --- \| --- \| --- \| --- \| |
| 77. Fainting | \| 1 \| 2 \| 3 \| 4 \| 5 \| \| --- \| --- \| --- \| --- \| --- \| |
| 78. Becoming nauseous | \| 1 \| 2 \| 3 \| 4 \| 5 \| \| --- \| --- \| --- \| --- \| --- \| |
| 79. Spiders | \| 1 \| 2 \| 3 \| 4 \| 5 \| \| --- \| --- \| --- \| --- \| --- \| |
| 80. Being in charge or responsible for decisions. | \| 1 \| 2 \| 3 \| 4 \| 5 \| \| --- \| --- \| --- \| --- \| --- \| |
| 81. Sight of knives or sharp objects | \| 1 \| 2 \| 3 \| 4 \| 5 \| \| --- \| --- \| --- \| --- \| --- \| |
| 82. Becoming mentally ill | \| 1 \| 2 \| 3 \| 4 \| 5 \| \| --- \| --- \| --- \| --- \| --- \| |
| 83. Being with a member of the opposite sex | \| 1 \| 2 \| 3 \| 4 \| 5 \| \| --- \| --- \| --- \| --- \| --- \| |
| 84. Taking written tests | \| 1 \| 2 \| 3 \| 4 \| 5 \| \| --- \| --- \| --- \| --- \| --- \| |
| 85. Being touched by others | \| 1 \| 2 \| 3 \| 4 \| 5 \| \| --- \| --- \| --- \| --- \| --- \| |
| 86. Feeling different from others | \| 1 \| 2 \| 3 \| 4 \| 5 \| \| --- \| --- \| --- \| --- \| --- \| |
| 87. A lull in conversation | \| 1 \| 2 \| 3 \| 4 \| 5 \| \| --- \| --- \| --- \| --- \| --- \| |

**Barrett Impulsivity Scale**

Rate yourself on the following items

1= Rarely/Never, 2= Occasionally, 3= Often, 4=Almost always/Always

|  | Rarely/Never \| Occasionally \| Often \| (Almost ) Always |
| --- | --- |
| 1. I plan tasks carefully | 1 2 3 4 |
| 2. I do things without thinking | 1 2 3 4 |
| 3. I make up my mind quickly | 1 2 3 4 |
| 4. I am happy-go-lucky | 1 2 3 4 |
| 5. I don't "pay attention" | 1 2 3 4 |
| 6. I have "racing" thoughts | 1 2 3 4 |
| 7. I plan trips well ahead of time | 1 2 3 4 |
| 8. I am self-controlled | 1 2 3 4 |
| 9. I concentrate easily | 1 2 3 4 |
| 10. I save money regularly | 1 2 3 4 |
| 11. I "squirm" at plays or talks | 1 2 3 4 |
| 12. I am a careful thinker | 1 2 3 4 |
| 13. I plan for job security | 1 2 3 4 |
| 14. I say things without thinking | 1 2 3 4 |
| 15. I like to think about complex problems | 1 2 3 4 |
| 16. I change jobs | 1 2 3 4 |
| 17. I act on impulse | 1 2 3 4 |
| 18. I get easily bored when solving thought problems | 1 2 3 4 |
| 19. I have regular health checks | 1 2 3 4 |
| 20. I act on the spur of the moment | 1 2 3 4 |
| 21. I am a steady thinker | 1 2 3 4 |
| 22. I change residences | 1 2 3 4 |
| 23. I buy things on impulse | 1 2 3 4 |
| 24. I can only things about one problem at a time | 1 2 3 4 |
| 25. I change hobbies. | 1 2 3 4 |
| 26. I walk and move fast | 1 2 3 4 |
| 27. I solve problems by trial and error | 1 2 3 4 |
| 28. I spend of charge more than I earn | 1 2 3 4 |
| 29. I talk fast | 1 2 3 4 |
| 30. I often have extraneous thoughts when thinking | 1 2 3 4 |
| 31. I am more interested in the present than the future | 1 2 3 4 |
| 32. I am restless at the theatre or lectures/classes | 1 2 3 4 |
| 33. I like puzzles | 1 2 3 4 |
| 33. I like puzzles | 1 2 3 4 |
| 34. I am future oriented | 1 2 3 4 |

**BEM SEX ROLE INVENTORY**

Rate yourself on each item, on a scale from 1 (never or almost never true) to 7 (almost always true).

|  | Never/Almost never | Almost  always |  | Never/Almost never | Almost  always |
| --- | --- | --- | --- | --- | --- |
| 1. self reliant | 1 2 3 4 5 6 7 | | 31. makes decisions easily | 1 2 3 4 5 6 7 | |
| 2. yielding | 1 2 3 4 5 6 7 | | 32. compassionate | 1 2 3 4 5 6 7 | |
| 3. helpful | 1 2 3 4 5 6 7 | | 33. sincere | 1 2 3 4 5 6 7 | |
| 4. defends own beliefs | 1 2 3 4 5 6 7 | | 34. self-sufficient | 1 2 3 4 5 6 7 | |
| 5. cheerful | 1 2 3 4 5 6 7 | | 35. eager to soothe hurt feelings | 1 2 3 4 5 6 7 | |
| 6. moody | 1 2 3 4 5 6 7 | | 36. conceited | 1 2 3 4 5 6 7 | |
| 7. independent | 1 2 3 4 5 6 7 | | 37. dominant | 1 2 3 4 5 6 7 | |
| 8. shy | 1 2 3 4 5 6 7 | | 38. soft spoken | 1 2 3 4 5 6 7 | |
| 9. conscientious | 1 2 3 4 5 6 7 | | 39. likable | 1 2 3 4 5 6 7 | |
| 10. athletic | 1 2 3 4 5 6 7 | | 40. masculine | 1 2 3 4 5 6 7 | |
| 11. affectionate | 1 2 3 4 5 6 7 | | 41. warm | 1 2 3 4 5 6 7 | |
| 12. theatrical | 1 2 3 4 5 6 7 | | 42. solemn | 1 2 3 4 5 6 7 | |
| 13. assertive | 1 2 3 4 5 6 7 | | 43. willing to take a stand | 1 2 3 4 5 6 7 | |
| 14. flatterable | 1 2 3 4 5 6 7 | | 44. tender | 1 2 3 4 5 6 7 | |
| 15. happy | 1 2 3 4 5 6 7 | | 45. friendly | 1 2 3 4 5 6 7 | |
| 16. strong personality | 1 2 3 4 5 6 7 | | 46. aggressive | 1 2 3 4 5 6 7 | |
| 17. loyal | 1 2 3 4 5 6 7 | | 47. gullible | 1 2 3 4 5 6 7 | |
| 18. unpredictable | 1 2 3 4 5 6 7 | | 48. inefficient | 1 2 3 4 5 6 7 | |
| 19. forceful | 1 2 3 4 5 6 7 | | 49. acts as a leader | 1 2 3 4 5 6 7 | |
| 20. feminine | 1 2 3 4 5 6 7 | | 50. childlike | 1 2 3 4 5 6 7 | |
| 21. reliable | 1 2 3 4 5 6 7 | | 51. adaptable | 1 2 3 4 5 6 7 | |
| 22. analytical | 1 2 3 4 5 6 7 | | 52. individualistic | 1 2 3 4 5 6 7 | |
| 23. sympathetic | 1 2 3 4 5 6 7 | | 53. does not use harsh language | 1 2 3 4 5 6 7 | |
| 24. jealous | 1 2 3 4 5 6 7 | | 54. unsystematic | 1 2 3 4 5 6 7 | |
| 25. leadership ability | 1 2 3 4 5 6 7 | | 55. competitive | 1 2 3 4 5 6 7 | |
| 26. sensitive to other's needs | 1 2 3 4 5 6 7 | | 56. loves children | 1 2 3 4 5 6 7 | |
| 27. truthful | 1 2 3 4 5 6 7 | | 57. tactful | 1 2 3 4 5 6 7 | |
| 28. willing to take risks | 1 2 3 4 5 6 7 | | 58. ambitious | 1 2 3 4 5 6 7 | |
| 29. understanding | 1 2 3 4 5 6 7 | | 59. gentle | 1 2 3 4 5 6 7 | |
| 30. secretive | 1 2 3 4 5 6 7 | | 60. conventional | 1 2 3 4 5 6 7 | |

**AddHealth Questions on Sexual Development**

**Have you experienced any of the following? If so, how old were you the first time?**

| **Have you…** |  | **If yes, how old were you?** |
| --- | --- | --- |
| Snogged / made out (kissing for a long time) | **Yes No** | ________ years _______ months |
| Felt someone’s breasts (or had your own felt) | **Yes No** | ________ years _______ months |
| Touched someone’s genital area (or had your own touched) | **Yes No** | ________ years _______ months |
| Had sexual intercourse? | **Yes No** | ________ years _______ months |
| Girls: First period  Boys: First started shaving | **Yes No** | ________ years _______ months |

**What is your sexuality? (please rate from 1 to 7 by circling a number)**

Homosexual 1 2 3 4 5 6 7 Heterosexual

**Family Background Questionnaire**

1. What was your living situation, during your childhood? (please tick one)

1. Lived with both parents throughout childhood ____
2. Parents separated and I remained with mother ____

(my age at separation=____)

1. My’s father died, leaving me with mother ____

(my age at death= ___)

1. My’s parents separated and I remained with father ____

(my age at separation=____)

1. My’s mother died, leaving me with father ____

(my age at death= ___)

1. I only ever lived with mother ____
2. I only ever lived with father ____

2. Do you have any step-parents/equivalent?

- 1. Had step-father since age ____ (If more than one, how many? ______)
  2. Had step-mother since age ___ (If more than one, how many? ______)

3. Whether they were separated or not, did your biological parents have a good or a bad quality relationship?  What was the quality of the relationship of the couple you lived with (if not both biological parents)? (1 = very poor quality, 9 = very good quality)

| **Parental unit** | **Before age of 6** | **From 6 until puberty** |
| --- | --- | --- |
| *Biological parents* | 1 2 3 4 5 6 7 8 9 | 1 2 3 4 5 6 7 8 9 |
| *Couple you lived with* | 1 2 3 4 5 6 7 8 9 | 1 2 3 4 5 6 7 8 9 |

4. What are the housing arrangements of your family?

|  |  |
| --- | --- |
| Number of bedrooms |  |
| Number of televisions |  |
| Number of cars |  |

5. What is your parents’ …

|  | age? | highest level of education? | Were/are their parents separated? |
| --- | --- | --- | --- |
| Mother: |  |  |  |
| Father: |  |  |  |
| Other primary carer: (m/f?) |  |  |  |

**Relationship Context Scale**

To what extent do the following terms describe your parent’s relationship with each other during your childhood (until you were 8)?

|  | Not at all true | Not quite true | Quite true | Absolutely true |
| --- | --- | --- | --- | --- |
| Contentment |  |  |  |  |
| Happiness |  |  |  |  |
| Love |  |  |  |  |
| Understanding |  |  |  |  |
| Partnership |  |  |  |  |

To what extent do the following terms describe you mother’s behaviour towards you during your childhood (until you were 8)?

|  | Not at all true | Not quite true | Quite true | Absolutely true |
| --- | --- | --- | --- | --- |
| Interest |  |  |  |  |
| Understanding |  |  |  |  |
| Acceptance |  |  |  |  |
| Love |  |  |  |  |
| Indifference |  |  |  |  |
| Rejection |  |  |  |  |

To what extent do the following terms describe you father’s behaviour towards you during your childhood (until you were 8)?

|  | Not at all true | Not quite true | Quite true | Absolutely true |
| --- | --- | --- | --- | --- |
| Interest |  |  |  |  |
| Understanding |  |  |  |  |
| Acceptance |  |  |  |  |
| Love |  |  |  |  |
| Indifference |  |  |  |  |
| Rejection |  |  |  |  |

How often did the following activities take place in your family during your childhood (until you were 8)?

|  | Never | Occasionally | Quite often | All the time |
| --- | --- | --- | --- | --- |
| Talking |  |  |  |  |
| Having fun |  |  |  |  |
| Avoiding someone |  |  |  |  |
| Touching |  |  |  |  |

How much of the following did you receive from your parents during your childhood (until you were 8)?

|  | Never | Occasionally | Quite often | All the time |
| --- | --- | --- | --- | --- |
| Care (provision for your material wellbeing) |  |  |  |  |
| Interest (in you) |  |  |  |  |
| Affection |  |  |  |  |

# SI 1.2 Relationships between age and other variables

# does age predict any of our outcome variables?

# make an agesquared variable to check for quadratic effects
masc_ageset$agesq <- masc_ageset$age * masc_ageset$age

# FSS_total has sig model but no sig parameters
fss_total.age <- lm(fss_total ~ age + agesq, data = masc_ageset,
 na.action = na.exclude)
summary(fss_total.age)

##
## Call:
## lm(formula = fss_total ~ age + agesq, data = masc_ageset, na.action = na.exclude)
##
## Residuals:
## Min 1Q Median 3Q Max
## -1.19099 -0.45362 -0.07007 0.36905 2.90993
##
## Coefficients:
## Estimate Std. Error t value Pr(>|t|)
## (Intercept) 4.644724 1.408251 3.298 0.00103 **
## age -0.213590 0.128518 -1.662 0.09704 .
## agesq 0.004293 0.002868 1.497 0.13492
## ---
## Signif. codes: 0 '***' 0.001 '**' 0.01 '*' 0.05 '.' 0.1 ' ' 1
##
## Residual standard error: 0.6419 on 608 degrees of freedom
## (80 observations deleted due to missingness)
## Multiple R-squared: 0.01376, Adjusted R-squared: 0.01051
## F-statistic: 4.241 on 2 and 608 DF, p-value: 0.01482

# age at menarche - nothing
periodyr.age <- lm(periodyr ~ age + agesq, data = masc_ageset,
 na.action = na.exclude)
summary(periodyr.age)

##
## Call:
## lm(formula = periodyr ~ age + agesq, data = masc_ageset, na.action = na.exclude)
##
## Residuals:
## Min 1Q Median 3Q Max
## -3.7579 -0.7203 0.2505 0.4970 4.4164
##
## Coefficients:
## Estimate Std. Error t value Pr(>|t|)
## (Intercept) 7.682971 3.361309 2.286 0.0227 *
## age 0.459359 0.306165 1.500 0.1341
## agesq -0.010395 0.006812 -1.526 0.1276
## ---
## Signif. codes: 0 '***' 0.001 '**' 0.01 '*' 0.05 '.' 0.1 ' ' 1
##
## Residual standard error: 1.449 on 533 degrees of freedom
## (155 observations deleted due to missingness)
## Multiple R-squared: 0.004559, Adjusted R-squared: 0.0008242
## F-statistic: 1.221 on 2 and 533 DF, p-value: 0.2959

# BSRI masc - nothing
bem_a.age <- lm(bsri_a ~ age + agesq, data = masc_ageset, na.action = na.exclude)
summary(bem_a.age)

##
## Call:
## lm(formula = bsri_a ~ age + agesq, data = masc_ageset, na.action = na.exclude)
##
## Residuals:
## Min 1Q Median 3Q Max
## -3.4574 -0.4441 0.0360 0.5167 2.5770
##
## Coefficients:
## Estimate Std. Error t value Pr(>|t|)
## (Intercept) 6.812807 1.833169 3.716 0.00022 ***
## age -0.236791 0.167090 -1.417 0.15694
## agesq 0.005843 0.003724 1.569 0.11719
## ---
## Signif. codes: 0 '***' 0.001 '**' 0.01 '*' 0.05 '.' 0.1 ' ' 1
##
## Residual standard error: 0.8598 on 628 degrees of freedom
## (60 observations deleted due to missingness)
## Multiple R-squared: 0.01131, Adjusted R-squared: 0.008159
## F-statistic: 3.591 on 2 and 628 DF, p-value: 0.02813

# BSRI fem - nothing
bem_b.age <- lm(bsri_b ~ age + agesq, data = masc_ageset, na.action = na.exclude)
summary(bem_b.age)

##
## Call:
## lm(formula = bsri_b ~ age + agesq, data = masc_ageset, na.action = na.exclude)
##
## Residuals:
## Min 1Q Median 3Q Max
## -3.7917 -0.4188 0.0329 0.5587 2.1896
##
## Coefficients:
## Estimate Std. Error t value Pr(>|t|)
## (Intercept) 5.647309 1.631986 3.460 0.000576 ***
## age -0.093946 0.148754 -0.632 0.527909
## agesq 0.002502 0.003315 0.755 0.450661
## ---
## Signif. codes: 0 '***' 0.001 '**' 0.01 '*' 0.05 '.' 0.1 ' ' 1
##
## Residual standard error: 0.7654 on 627 degrees of freedom
## (61 observations deleted due to missingness)
## Multiple R-squared: 0.00594, Adjusted R-squared: 0.002769
## F-statistic: 1.873 on 2 and 627 DF, p-value: 0.1545

# AQ anger - nothing
ang.age <- lm(agg_ang ~ age + agesq, data = masc_ageset, na.action = na.exclude)
summary(ang.age)

##
## Call:
## lm(formula = agg_ang ~ age + agesq, data = masc_ageset, na.action = na.exclude)
##
## Residuals:
## Min 1Q Median 3Q Max
## -1.61367 -0.61367 0.01037 0.60176 2.35133
##
## Coefficients:
## Estimate Std. Error t value Pr(>|t|)
## (Intercept) 4.313950 1.804210 2.391 0.0171 *
## age -0.166439 0.164366 -1.013 0.3117
## agesq 0.003832 0.003658 1.048 0.2952
## ---
## Signif. codes: 0 '***' 0.001 '**' 0.01 '*' 0.05 '.' 0.1 ' ' 1
##
## Residual standard error: 0.8191 on 563 degrees of freedom
## (125 observations deleted due to missingness)
## Multiple R-squared: 0.002352, Adjusted R-squared: -0.001192
## F-statistic: 0.6638 on 2 and 563 DF, p-value: 0.5153

# AQ hostility - nothing
host.age <- lm(agg_host ~ age + agesq, data = masc_ageset, na.action = na.exclude)
summary(host.age)

##
## Call:
## lm(formula = agg_host ~ age + agesq, data = masc_ageset, na.action = na.exclude)
##
## Residuals:
## Min 1Q Median 3Q Max
## -1.89136 -0.62614 0.02123 0.64917 2.29688
##
## Coefficients:
## Estimate Std. Error t value Pr(>|t|)
## (Intercept) 2.8475850 1.8750630 1.519 0.129
## age 0.0160962 0.1707948 0.094 0.925
## agesq -0.0007591 0.0038002 -0.200 0.842
##
## Residual standard error: 0.8503 on 560 degrees of freedom
## (128 observations deleted due to missingness)
## Multiple R-squared: 0.004295, Adjusted R-squared: 0.0007393
## F-statistic: 1.208 on 2 and 560 DF, p-value: 0.2996

# AQ physical aggression - weak variable coeffs, nonsig model
phys.age <- lm(agg_phys ~ age + agesq, data = masc_ageset, na.action = na.exclude)
summary(phys.age)

##
## Call:
## lm(formula = agg_phys ~ age + agesq, data = masc_ageset, na.action = na.exclude)
##
## Residuals:
## Min 1Q Median 3Q Max
## -1.4417 -0.7015 -0.1117 0.6506 2.3383
##
## Coefficients:
## Estimate Std. Error t value Pr(>|t|)
## (Intercept) 6.211901 1.881719 3.301 0.00102 **
## age -0.341323 0.171443 -1.991 0.04698 *
## agesq 0.007326 0.003815 1.920 0.05534 .
## ---
## Signif. codes: 0 '***' 0.001 '**' 0.01 '*' 0.05 '.' 0.1 ' ' 1
##
## Residual standard error: 0.8547 on 563 degrees of freedom
## (125 observations deleted due to missingness)
## Multiple R-squared: 0.008636, Adjusted R-squared: 0.005114
## F-statistic: 2.452 on 2 and 563 DF, p-value: 0.08702

# AQ verbal aggression - weak variable coeffs, nonsig model
verb.age <- lm(agg_verb ~ age + agesq, data = masc_ageset, na.action = na.exclude)
summary(verb.age)

##
## Call:
## lm(formula = agg_verb ~ age + agesq, data = masc_ageset, na.action = na.exclude)
##
## Residuals:
## Min 1Q Median 3Q Max
## -2.02392 -0.62392 -0.02392 0.57608 2.13372
##
## Coefficients:
## Estimate Std. Error t value Pr(>|t|)
## (Intercept) 6.740557 1.863145 3.618 0.000324 ***
## age -0.343398 0.169709 -2.023 0.043501 *
## agesq 0.007607 0.003776 2.014 0.044439 *
## ---
## Signif. codes: 0 '***' 0.001 '**' 0.01 '*' 0.05 '.' 0.1 ' ' 1
##
## Residual standard error: 0.8449 on 560 degrees of freedom
## (128 observations deleted due to missingness)
## Multiple R-squared: 0.007265, Adjusted R-squared: 0.00372
## F-statistic: 2.049 on 2 and 560 DF, p-value: 0.1298

# BIS motor- nothing
BISmot.age <- lm(bis_motor ~ age + agesq, data = masc_ageset,
 na.action = na.exclude)
summary(BISmot.age)

##
## Call:
## lm(formula = bis_motor ~ age + agesq, data = masc_ageset, na.action = na.exclude)
##
## Residuals:
## Min 1Q Median 3Q Max
## -1.3375 -0.3362 -0.0375 0.3567 1.6898
##
## Coefficients:
## Estimate Std. Error t value Pr(>|t|)
## (Intercept) 1.424596 1.060997 1.343 0.180
## age 0.097415 0.096705 1.007 0.314
## agesq -0.002598 0.002155 -1.206 0.228
##
## Residual standard error: 0.494 on 619 degrees of freedom
## (69 observations deleted due to missingness)
## Multiple R-squared: 0.01555, Adjusted R-squared: 0.01237
## F-statistic: 4.888 on 2 and 619 DF, p-value: 0.007827

# BIS attention - nothing
BISatt.age <- lm(bis_attention ~ age + agesq, data = masc_ageset,
 na.action = na.exclude)
summary(BISatt.age)

##
## Call:
## lm(formula = bis_attention ~ age + agesq, data = masc_ageset,
## na.action = na.exclude)
##
## Residuals:
## Min 1Q Median 3Q Max
## -1.28282 -0.28282 -0.03282 0.23096 1.21718
##
## Coefficients:
## Estimate Std. Error t value Pr(>|t|)
## (Intercept) 1.404549 0.912906 1.539 0.124
## age 0.099205 0.083204 1.192 0.234
## agesq -0.002399 0.001854 -1.294 0.196
##
## Residual standard error: 0.4239 on 615 degrees of freedom
## (73 observations deleted due to missingness)
## Multiple R-squared: 0.006171, Adjusted R-squared: 0.002939
## F-statistic: 1.909 on 2 and 615 DF, p-value: 0.1491

# BIS planning - nothing
BISplan.age <- lm(bis_planning ~ age + agesq, data = masc_ageset,
 na.action = na.exclude)
summary(BISplan.age)

##
## Call:
## lm(formula = bis_planning ~ age + agesq, data = masc_ageset,
## na.action = na.exclude)
##
## Residuals:
## Min 1Q Median 3Q Max
## -1.11416 -0.27572 -0.02379 0.21584 1.23153
##
## Coefficients:
## Estimate Std. Error t value Pr(>|t|)
## (Intercept) 2.424e+00 8.195e-01 2.958 0.00321 **
## age -3.751e-03 7.470e-02 -0.050 0.95997
## agesq 3.727e-05 1.664e-03 0.022 0.98214
## ---
## Signif. codes: 0 '***' 0.001 '**' 0.01 '*' 0.05 '.' 0.1 ' ' 1
##
## Residual standard error: 0.3814 on 618 degrees of freedom
## (70 observations deleted due to missingness)
## Multiple R-squared: 0.0002739, Adjusted R-squared: -0.002961
## F-statistic: 0.08467 on 2 and 618 DF, p-value: 0.9188

# SI 1.3 Checking income as a predictor of our measures of interest

is father absence associated with lower income (regression other way round bc factors)? Not really - but we add income to models here for completeness

# age at 1st sex - nothing
income.sex_yr <- lm(sex_yr ~ income, data = masc_ageset)
summary(income.sex_yr)

##
## Call:
## lm(formula = sex_yr ~ income, data = masc_ageset)
##
## Residuals:
## Min 1Q Median 3Q Max
## -7.6014 -1.5789 0.3761 1.3986 14.3761
##
## Coefficients:
## Estimate Std. Error t value Pr(>|t|)
## (Intercept) 16.53396 0.36567 45.215 <2e-16 ***
## income 0.02248 0.13424 0.167 0.867
## ---
## Signif. codes: 0 '***' 0.001 '**' 0.01 '*' 0.05 '.' 0.1 ' ' 1
##
## Residual standard error: 2.15 on 440 degrees of freedom
## (249 observations deleted due to missingness)
## Multiple R-squared: 6.375e-05, Adjusted R-squared: -0.002209
## F-statistic: 0.02805 on 1 and 440 DF, p-value: 0.8671

# age at menarche - nothing
income.periodyr <- lm(periodyr ~ income, data = masc_ageset)
summary(income.periodyr)

##
## Call:
## lm(formula = periodyr ~ income, data = masc_ageset)
##
## Residuals:
## Min 1Q Median 3Q Max
## -3.6394 -0.6394 0.3606 0.3825 4.3825
##
## Coefficients:
## Estimate Std. Error t value Pr(>|t|)
## (Intercept) 12.66129 0.22314 56.741 <2e-16 ***
## income -0.01094 0.08267 -0.132 0.895
## ---
## Signif. codes: 0 '***' 0.001 '**' 0.01 '*' 0.05 '.' 0.1 ' ' 1
##
## Residual standard error: 1.454 on 532 degrees of freedom
## (157 observations deleted due to missingness)
## Multiple R-squared: 3.291e-05, Adjusted R-squared: -0.001847
## F-statistic: 0.01751 on 1 and 532 DF, p-value: 0.8948

# reactivity - nothing
income.reactivity <- lm(reactivity ~ income, data = masc_ageset)
summary(income.reactivity)

##
## Call:
## lm(formula = reactivity ~ income, data = masc_ageset)
##
## Residuals:
## Min 1Q Median 3Q Max
## -2.41145 -0.75157 -0.02093 0.68576 3.08120
##
## Coefficients:
## Estimate Std. Error t value Pr(>|t|)
## (Intercept) -0.019544 0.151908 -0.129 0.898
## income 0.001919 0.056423 0.034 0.973
##
## Residual standard error: 0.9958 on 521 degrees of freedom
## (168 observations deleted due to missingness)
## Multiple R-squared: 2.22e-06, Adjusted R-squared: -0.001917
## F-statistic: 0.001157 on 1 and 521 DF, p-value: 0.9729

# masculinity - nothing
income.masculinity <- lm(masculinity ~ income, data = masc_ageset)
summary(income.masculinity)

##
## Call:
## lm(formula = masculinity ~ income, data = masc_ageset)
##
## Residuals:
## Min 1Q Median 3Q Max
## -3.2554 -0.6304 -0.0392 0.6429 3.3862
##
## Coefficients:
## Estimate Std. Error t value Pr(>|t|)
## (Intercept) -0.13099 0.14796 -0.885 0.376
## income 0.04630 0.05496 0.842 0.400
##
## Residual standard error: 0.9699 on 521 degrees of freedom
## (168 observations deleted due to missingness)
## Multiple R-squared: 0.00136, Adjusted R-squared: -0.0005563
## F-statistic: 0.7098 on 1 and 521 DF, p-value: 0.3999

# poorer relationships (strongly) associated with lower income
income.quality <- lm(FRQ ~ income, data = masc_ageset)
summary(income.quality)

##
## Call:
## lm(formula = FRQ ~ income, data = masc_ageset)
##
## Residuals:
## Min 1Q Median 3Q Max
## -3.2832 -0.7023 0.1631 0.8217 1.6326
##
## Coefficients:
## Estimate Std. Error t value Pr(>|t|)
## (Intercept) 0.9298 0.1317 7.059 4.4e-12 ***
## income -0.3571 0.0488 -7.318 7.6e-13 ***
## ---
## Signif. codes: 0 '***' 0.001 '**' 0.01 '*' 0.05 '.' 0.1 ' ' 1
##
## Residual standard error: 0.9608 on 639 degrees of freedom
## (50 observations deleted due to missingness)
## Multiple R-squared: 0.07732, Adjusted R-squared: 0.07588
## F-statistic: 53.55 on 1 and 639 DF, p-value: 7.603e-13

etasq(income.quality)

## Partial eta^2
## income 0.0773197
## Residuals NA

# father absence before age 12 (strongly) associated with lower income. No difference between father absent after age 12 and father present

masc$fath_ab <- as.factor(masc$fath_ab) # make sure fath_ab is a factor not continuous
father.absence.income <- lm(income ~ fath_ab, data = masc)
summary(father.absence.income)

##
## Call:
## lm(formula = income ~ fath_ab, data = masc)
##
## Residuals:
## Min 1Q Median 3Q Max
## -1.92035 -0.47772 0.07965 0.52228 1.52228
##
## Coefficients:
## Estimate Std. Error t value Pr(>|t|)
## (Intercept) 2.47772 0.03751 66.064 < 2e-16 ***
## fath_ab0.5 0.08160 0.10506 0.777 0.438
## fath_ab1 0.44263 0.08022 5.518 5.21e-08 ***
## ---
## Signif. codes: 0 '***' 0.001 '**' 0.01 '*' 0.05 '.' 0.1 ' ' 1
##
## Residual standard error: 0.7538 on 573 degrees of freedom
## (2 observations deleted due to missingness)
## Multiple R-squared: 0.05048, Adjusted R-squared: 0.04717
## F-statistic: 15.23 on 2 and 573 DF, p-value: 3.586e-07

etasq(father.absence.income)

## Partial eta^2
## fath_ab 0.05048277
## Residuals NA

# SI 1.4 Re-analysis with income added to models

Although income is not a predictor of our outcome measures, we present here each analysis from the paper with income added to the model. We will present the text of the analysis from the paper and then the R output for the reanalysis with income for each analysis.

“When women were classified as father present and father *ever* absent as the men were, there was a weak but significant effect of father absence, such that those who had ever experienced father absence were more reactive than those who remained father present”

masc$f_ab_ever <- as.factor(masc$f_ab_ever) # make sure f_ab_ever is a factor not continuous
father.absence.reactivity2f.income <- lm(reactivity ~ f_ab_ever *
 country + income, data = masc[masc$gender == "female", ])
summary(father.absence.reactivity2f.income)

##
## Call:
## lm(formula = reactivity ~ f_ab_ever * country + income, data = masc[masc$gender ==
## "female", ])
##
## Residuals:
## Min 1Q Median 3Q Max
## -2.30314 -0.70354 -0.05489 0.66937 3.10206
##
## Coefficients:
## Estimate Std. Error t value Pr(>|t|)
## (Intercept) -0.21531 0.18197 -1.183 0.23746
## f_ab_ever1 0.28748 0.14033 2.049 0.04119 *
## countryUnited State -0.49698 0.15408 -3.225 0.00137 **
## income 0.08937 0.06810 1.312 0.19020
## f_ab_ever1:countryUnited State -0.12606 0.24370 -0.517 0.60526
## ---
## Signif. codes: 0 '***' 0.001 '**' 0.01 '*' 0.05 '.' 0.1 ' ' 1
##
## Residual standard error: 0.9777 on 380 degrees of freedom
## (107 observations deleted due to missingness)
## Multiple R-squared: 0.05785, Adjusted R-squared: 0.04793
## F-statistic: 5.833 on 4 and 380 DF, p-value: 0.0001451

etasq(father.absence.reactivity2f.income)

## Partial eta^2
## f_ab_ever 0.0118577694
## country 0.0522757555
## income 0.0045117694
## f_ab_ever:country 0.0007036742
## Residuals NA

“However, when father-absent women were coded as father absent before or after 12, neither of these groups had higher reactivity than the father present group”

father.absence.reactivityf.income <- lm(reactivity ~ fath_ab *
 country + income, data = masc[masc$gender == "female", ])
summary(father.absence.reactivityf.income)

##
## Call:
## lm(formula = reactivity ~ fath_ab * country + income, data = masc[masc$gender ==
## "female", ])
##
## Residuals:
## Min 1Q Median 3Q Max
## -2.31484 -0.68764 -0.05224 0.66601 3.09819
##
## Coefficients:
## Estimate Std. Error t value Pr(>|t|)
## (Intercept) -0.19579 0.18355 -1.067 0.2868
## fath_ab0.5 0.27087 0.19617 1.381 0.1681
## fath_ab1 0.30261 0.18054 1.676 0.0945 .
## countryUnited State -0.49671 0.15432 -3.219 0.0014 **
## income 0.08155 0.06876 1.186 0.2364
## fath_ab0.5:countryUnited State -0.29952 0.34923 -0.858 0.3916
## fath_ab1:countryUnited State -0.05063 0.28571 -0.177 0.8594
## ---
## Signif. codes: 0 '***' 0.001 '**' 0.01 '*' 0.05 '.' 0.1 ' ' 1
##
## Residual standard error: 0.9791 on 378 degrees of freedom
## (107 observations deleted due to missingness)
## Multiple R-squared: 0.05996, Adjusted R-squared: 0.04504
## F-statistic: 4.019 on 6 and 378 DF, p-value: 0.0006459

etasq(father.absence.reactivityf.income)

## Partial eta^2
## fath_ab 0.012865738
## country 0.053145354
## income 0.003707022
## fath_ab:country 0.001944249
## Residuals NA

“When women were classified as father present and father *ever* absent as the men were, there was no effect of having ever experienced father absence on masculinity scores”

father.absence.masculinity2f.income <- lm(masculinity ~ f_ab_ever *
 country + income, data = masc[masc$gender == "female", ])
summary(father.absence.masculinity2f.income)

##
## Call:
## lm(formula = masculinity ~ f_ab_ever * country + income, data = masc[masc$gender ==
## "female", ])
##
## Residuals:
## Min 1Q Median 3Q Max
## -3.3902 -0.6158 -0.0622 0.6020 2.4989
##
## Coefficients:
## Estimate Std. Error t value Pr(>|t|)
## (Intercept) -0.13439 0.16988 -0.791 0.42941
## f_ab_ever1 0.03634 0.13101 0.277 0.78161
## countryUnited State 0.44333 0.14385 3.082 0.00221 **
## income -0.01190 0.06358 -0.187 0.85164
## f_ab_ever1:countryUnited State -0.34377 0.22751 -1.511 0.13162
## ---
## Signif. codes: 0 '***' 0.001 '**' 0.01 '*' 0.05 '.' 0.1 ' ' 1
##
## Residual standard error: 0.9127 on 380 degrees of freedom
## (107 observations deleted due to missingness)
## Multiple R-squared: 0.02518, Adjusted R-squared: 0.01492
## F-statistic: 2.454 on 4 and 380 DF, p-value: 0.04546

etasq(father.absence.masculinity2f.income)

## Partial eta^2
## f_ab_ever 1.334636e-03
## country 1.942399e-02
## income 9.216089e-05
## f_ab_ever:country 5.972261e-03
## Residuals NA

“When women were coded as either father absent before 12, father absent after 12, or never father absent, neither group had higher masculinity than the father present group … but there was a significant interaction between country and father absence before 12”

father.absence.masculinityf.income <- lm(masculinity ~ fath_ab *
 country + income, data = masc[masc$gender == "female", ])
summary(father.absence.masculinityf.income)

##
## Call:
## lm(formula = masculinity ~ fath_ab * country + income, data = masc[masc$gender ==
## "female", ])
##
## Residuals:
## Min 1Q Median 3Q Max
## -3.4030 -0.6153 -0.0494 0.5995 2.5262
##
## Coefficients:
## Estimate Std. Error t value Pr(>|t|)
## (Intercept) -0.11352 0.17025 -0.667 0.50531
## fath_ab0.5 -0.26919 0.18195 -1.479 0.13986
## fath_ab1 0.28950 0.16746 1.729 0.08466 .
## countryUnited State 0.44362 0.14313 3.099 0.00208 **
## income -0.02026 0.06378 -0.318 0.75094
## fath_ab0.5:countryUnited State -0.06164 0.32392 -0.190 0.84919
## fath_ab1:countryUnited State -0.58185 0.26501 -2.196 0.02873 *
## ---
## Signif. codes: 0 '***' 0.001 '**' 0.01 '*' 0.05 '.' 0.1 ' ' 1
##
## Residual standard error: 0.9082 on 378 degrees of freedom
## (107 observations deleted due to missingness)
## Multiple R-squared: 0.03995, Adjusted R-squared: 0.02471
## F-statistic: 2.621 on 6 and 378 DF, p-value: 0.01677

etasq(father.absence.masculinityf.income)

## Partial eta^2
## fath_ab 0.0096736235
## country 0.0176346930
## income 0.0002668372
## fath_ab:country 0.0128962026
## Residuals NA

“We therefore ran analyses separately for the US and Australia. In the US, the effect of father absence before 12 was clearly non-significant… whereas the effect in Australia of father absence before 12 was in the predicted direction (i.e. father-absent women were more masculine), although very small in size and with a two-tailed p-value of .06”

# US women

father.absence.masculinity.US.income <- lm(masculinity ~ fath_ab +
 income, data = masc[(masc$gender == "female") & (masc$country ==
 "United State"), ])
summary(father.absence.masculinity.US.income)

##
## Call:
## lm(formula = masculinity ~ fath_ab + income, data = masc[(masc$gender ==
## "female") & (masc$country == "United State"), ])
##
## Residuals:
## Min 1Q Median 3Q Max
## -3.15200 -0.63582 -0.02221 0.67149 2.44972
##
## Coefficients:
## Estimate Std. Error t value Pr(>|t|)
## (Intercept) -0.08481 0.37642 -0.225 0.822
## fath_ab0.5 -0.33127 0.31543 -1.050 0.296
## fath_ab1 -0.37924 0.25127 -1.509 0.135
## income 0.14370 0.13596 1.057 0.293
##
## Residual standard error: 1.069 on 93 degrees of freedom
## (71 observations deleted due to missingness)
## Multiple R-squared: 0.03313, Adjusted R-squared: 0.00194
## F-statistic: 1.062 on 3 and 93 DF, p-value: 0.3691

etasq(father.absence.masculinity.US.income)

## Partial eta^2
## fath_ab 0.02800580
## income 0.01186875
## Residuals NA

# Australian women

father.absence.masculinity.Aus.income <- lm(masculinity ~ fath_ab +
 income, data = masc[(masc$gender == "female") & (masc$country ==
 "Australia"), ])
summary(father.absence.masculinity.Aus.income)

##
## Call:
## lm(formula = masculinity ~ fath_ab + income, data = masc[(masc$gender ==
## "female") & (masc$country == "Australia"), ])
##
## Residuals:
## Min 1Q Median 3Q Max
## -2.76809 -0.57970 -0.02499 0.55962 2.42028
##
## Coefficients:
## Estimate Std. Error t value Pr(>|t|)
## (Intercept) 0.06594 0.18646 0.354 0.7239
## fath_ab0.5 -0.26887 0.16943 -1.587 0.1137
## fath_ab1 0.30251 0.15609 1.938 0.0536 .
## income -0.09217 0.07124 -1.294 0.1968
## ---
## Signif. codes: 0 '***' 0.001 '**' 0.01 '*' 0.05 '.' 0.1 ' ' 1
##
## Residual standard error: 0.8457 on 284 degrees of freedom
## (36 observations deleted due to missingness)
## Multiple R-squared: 0.02864, Adjusted R-squared: 0.01838
## F-statistic: 2.791 on 3 and 284 DF, p-value: 0.0408

etasq(father.absence.masculinity.Aus.income)

## Partial eta^2
## fath_ab 0.024433694
## income 0.005860286
## Residuals NA

“Australian men showed no effect of father absence for reactivity scores…”

father.absence.reactivity2m.income <- lm(reactivity ~ f_ab_ever +
 income, data = masc[masc$gender == "male", ])
summary(father.absence.reactivity2m.income)

##
## Call:
## lm(formula = reactivity ~ f_ab_ever + income, data = masc[masc$gender ==
## "male", ])
##
## Residuals:
## Min 1Q Median 3Q Max
## -1.76159 -0.48273 -0.03473 0.50794 2.17339
##
## Coefficients:
## Estimate Std. Error t value Pr(>|t|)
## (Intercept) 0.7808 0.3638 2.146 0.0357 *
## f_ab_ever1 0.2514 0.2727 0.922 0.3601
## income -0.2802 0.1367 -2.050 0.0445 *
## ---
## Signif. codes: 0 '***' 0.001 '**' 0.01 '*' 0.05 '.' 0.1 ' ' 1
##
## Residual standard error: 0.9361 on 63 degrees of freedom
## (20 observations deleted due to missingness)
## Multiple R-squared: 0.06735, Adjusted R-squared: 0.03774
## F-statistic: 2.275 on 2 and 63 DF, p-value: 0.1112

etasq(father.absence.reactivity2m.income)

## Partial eta^2
## f_ab_ever 0.01331140
## income 0.06254535
## Residuals NA

“…or masculinity scores.”

father.absence.masculinity2m.income <- lm(masculinity ~ f_ab_ever +
 income, data = masc[masc$gender == "male", ])
summary(father.absence.masculinity2m.income)

##
## Call:
## lm(formula = masculinity ~ f_ab_ever + income, data = masc[masc$gender ==
## "male", ])
##
## Residuals:
## Min 1Q Median 3Q Max
## -2.1714 -0.8294 -0.1267 0.8135 3.2239
##
## Coefficients:
## Estimate Std. Error t value Pr(>|t|)
## (Intercept) 0.06194 0.43891 0.141 0.888
## f_ab_ever1 0.16800 0.32893 0.511 0.611
## income 0.03864 0.16485 0.234 0.815
##
## Residual standard error: 1.129 on 63 degrees of freedom
## (20 observations deleted due to missingness)
## Multiple R-squared: 0.005774, Adjusted R-squared: -0.02579
## F-statistic: 0.1829 on 2 and 63 DF, p-value: 0.8333

etasq(father.absence.masculinity2m.income)

## Partial eta^2
## f_ab_ever 0.0041236875
## income 0.0008712384
## Residuals NA

**Ratings of parents and factor scores**

“Overall, participant perceptions of parental marital quality were negatively related to reactivity scores, such that participants whose parents had a better quality relationship were less reactive; this was true for both male…”

quality.reactivity.m.income <- lm(reactivity ~ FRQ * country +
 income, data = masc_ageset[masc_ageset$gender == "male",
 ])
etasq(quality.reactivity.m.income)

## Partial eta^2
## FRQ 0.208236942
## country 0.028589595
## income 0.016971698
## FRQ:country 0.005765382
## Residuals NA

summary(quality.reactivity.m.income)

##
## Call:
## lm(formula = reactivity ~ FRQ * country + income, data = masc_ageset[masc_ageset$gender ==
## "male", ])
##
## Residuals:
## Min 1Q Median 3Q Max
## -1.92249 -0.64696 0.00588 0.47522 2.47098
##
## Coefficients:
## Estimate Std. Error t value Pr(>|t|)
## (Intercept) 0.5777 0.3106 1.860 0.0664 .
## FRQ -0.5236 0.1137 -4.605 1.43e-05 ***
## countryUnited State -0.4421 0.2556 -1.730 0.0873 .
## income -0.1424 0.1176 -1.211 0.2291
## FRQ:countryUnited State 0.2016 0.2872 0.702 0.4846
## ---
## Signif. codes: 0 '***' 0.001 '**' 0.01 '*' 0.05 '.' 0.1 ' ' 1
##
## Residual standard error: 0.8936 on 85 degrees of freedom
## (43 observations deleted due to missingness)
## Multiple R-squared: 0.266, Adjusted R-squared: 0.2314
## F-statistic: 7.701 on 4 and 85 DF, p-value: 2.412e-05

“…and female participants.”

quality.reactivity.f.income <- lm(reactivity ~ FRQ * country +
 income, data = masc_ageset[masc_ageset$gender == "female",
 ])
etasq(quality.reactivity.f.income)

## Partial eta^2
## FRQ 0.0772919507
## country 0.0353116850
## income 0.0001807806
## FRQ:country 0.0032125491
## Residuals NA

summary(quality.reactivity.f.income)

##
## Call:
## lm(formula = reactivity ~ FRQ * country + income, data = masc_ageset[masc_ageset$gender ==
## "female", ])
##
## Residuals:
## Min 1Q Median 3Q Max
## -2.4814 -0.6507 -0.0035 0.6412 3.1995
##
## Coefficients:
## Estimate Std. Error t value Pr(>|t|)
## (Intercept) 0.05158 0.17123 0.301 0.763407
## FRQ -0.32450 0.06012 -5.398 1.16e-07 ***
## countryUnited State -0.39215 0.10568 -3.711 0.000236 ***
## income 0.01723 0.06398 0.269 0.787861
## FRQ:countryUnited State 0.11352 0.09985 1.137 0.256288
## ---
## Signif. codes: 0 '***' 0.001 '**' 0.01 '*' 0.05 '.' 0.1 ' ' 1
##
## Residual standard error: 0.9348 on 401 degrees of freedom
## (152 observations deleted due to missingness)
## Multiple R-squared: 0.1034, Adjusted R-squared: 0.09449
## F-statistic: 11.56 on 4 and 401 DF, p-value: 6.767e-09

“Participant perceptions of parental marital quality did not predict masculinity scores for men…”

quality.masculinity.m.income <- lm(masculinity ~ FRQ * country +
 income, data = masc_ageset[masc_ageset$gender == "male",
 ])
summary(quality.masculinity.m.income)

##
## Call:
## lm(formula = masculinity ~ FRQ * country + income, data = masc_ageset[masc_ageset$gender ==
## "male", ])
##
## Residuals:
## Min 1Q Median 3Q Max
## -2.5173 -0.7320 -0.1296 0.7221 3.2616
##
## Coefficients:
## Estimate Std. Error t value Pr(>|t|)
## (Intercept) 0.06729 0.37130 0.181 0.8566
## FRQ 0.19162 0.13591 1.410 0.1622
## countryUnited State 0.57515 0.30552 1.882 0.0632 .
## income 0.03177 0.14053 0.226 0.8217
## FRQ:countryUnited State -0.37788 0.34330 -1.101 0.2741
## ---
## Signif. codes: 0 '***' 0.001 '**' 0.01 '*' 0.05 '.' 0.1 ' ' 1
##
## Residual standard error: 1.068 on 85 degrees of freedom
## (43 observations deleted due to missingness)
## Multiple R-squared: 0.06309, Adjusted R-squared: 0.019
## F-statistic: 1.431 on 4 and 85 DF, p-value: 0.2308

etasq(quality.masculinity.m.income)

## Partial eta^2
## FRQ 0.0128841817
## country 0.0278348483
## income 0.0006009629
## FRQ:country 0.0140540889
## Residuals NA

“…or women”

quality.masculinity.f.income <- lm(masculinity ~ FRQ * country +
 income, data = masc_ageset[masc_ageset$gender == "female",
 ])
summary(quality.masculinity.f.income)

##
## Call:
## lm(formula = masculinity ~ FRQ * country + income, data = masc_ageset[masc_ageset$gender ==
## "female", ])
##
## Residuals:
## Min 1Q Median 3Q Max
## -3.1924 -0.6161 -0.0565 0.6430 2.6044
##
## Coefficients:
## Estimate Std. Error t value Pr(>|t|)
## (Intercept) -0.31120 0.17094 -1.821 0.06942 .
## FRQ 0.05980 0.06001 0.996 0.31966
## countryUnited State 0.34796 0.10550 3.298 0.00106 **
## income 0.05763 0.06387 0.902 0.36749
## FRQ:countryUnited State 0.09341 0.09968 0.937 0.34927
## ---
## Signif. codes: 0 '***' 0.001 '**' 0.01 '*' 0.05 '.' 0.1 ' ' 1
##
## Residual standard error: 0.9332 on 401 degrees of freedom
## (152 observations deleted due to missingness)
## Multiple R-squared: 0.03402, Adjusted R-squared: 0.02438
## F-statistic: 3.53 on 4 and 401 DF, p-value: 0.007579

etasq(quality.masculinity.f.income)

## Partial eta^2
## FRQ 0.008685349
## country 0.025292088
## income 0.002025782
## FRQ:country 0.002185132
## Residuals NA

**Reproductive outcomes**

“Father absence did not predict age of first coitus in Australian men”

father.absence.sex.m.income <- lm(sex_yr ~ f_ab_ever + income,
 data = masc[masc$gender == "male", ])
summary(father.absence.sex.m.income)

##
## Call:
## lm(formula = sex_yr ~ f_ab_ever + income, data = masc[masc$gender ==
## "male", ])
##
## Residuals:
## Min 1Q Median 3Q Max
## -4.510 -1.322 0.490 1.490 4.555
##
## Coefficients:
## Estimate Std. Error t value Pr(>|t|)
## (Intercept) 16.31578 1.01425 16.087 <2e-16 ***
## f_ab_ever1 0.50851 0.68350 0.744 0.461
## income 0.06474 0.39293 0.165 0.870
## ---
## Signif. codes: 0 '***' 0.001 '**' 0.01 '*' 0.05 '.' 0.1 ' ' 1
##
## Residual standard error: 2.138 on 47 degrees of freedom
## (36 observations deleted due to missingness)
## Multiple R-squared: 0.01346, Adjusted R-squared: -0.02852
## F-statistic: 0.3207 on 2 and 47 DF, p-value: 0.7272

etasq(father.absence.sex.m.income)

## Partial eta^2
## f_ab_ever 0.0116396911
## income 0.0005771841
## Residuals NA

“”Women who were father absent before age 12 experienced first coitus significantly earlier than father present women in the United States...”

father.absence.sex.US.income <- lm(sex_yr ~ fath_ab + income,
 data = masc[(masc$gender == "female") & (masc$country ==
 "United State"), ])
summary(father.absence.sex.US.income)

##
## Call:
## lm(formula = sex_yr ~ fath_ab + income, data = masc[(masc$gender ==
## "female") & (masc$country == "United State"), ])
##
## Residuals:
## Min 1Q Median 3Q Max
## -7.9227 -1.5148 0.0773 1.4347 7.2831
##
## Coefficients:
## Estimate Std. Error t value Pr(>|t|)
## (Intercept) 17.5291 0.6589 26.603 < 2e-16 ***
## fath_ab0.5 -0.1467 0.5989 -0.245 0.80689
## fath_ab1 -1.4079 0.4227 -3.330 0.00113 **
## income -0.2021 0.2436 -0.830 0.40814
## ---
## Signif. codes: 0 '***' 0.001 '**' 0.01 '*' 0.05 '.' 0.1 ' ' 1
##
## Residual standard error: 2.174 on 130 degrees of freedom
## (34 observations deleted due to missingness)
## Multiple R-squared: 0.1067, Adjusted R-squared: 0.08607
## F-statistic: 5.175 on 3 and 130 DF, p-value: 0.00207

etasq(father.absence.sex.US.income)

## Partial eta^2
## fath_ab 0.084087609
## income 0.005269425
## Residuals NA

“…This effect was not statistically significant in Australia…”

father.absence.sex.Aus.income <- lm(sex_yr ~ fath_ab + income,
 data = masc[(masc$gender == "female") & (masc$country ==
 "Australia"), ])
summary(father.absence.sex.Aus.income)

##
## Call:
## lm(formula = sex_yr ~ fath_ab + income, data = masc[(masc$gender ==
## "female") & (masc$country == "Australia"), ])
##
## Residuals:
## Min 1Q Median 3Q Max
## -3.3945 -1.1829 -0.1142 0.8858 13.0932
##
## Coefficients:
## Estimate Std. Error t value Pr(>|t|)
## (Intercept) 14.73633 0.60955 24.176 < 2e-16 ***
## fath_ab0.5 0.28029 0.48714 0.575 0.565781
## fath_ab1 0.06875 0.43693 0.157 0.875164
## income 0.79261 0.23293 3.403 0.000831 ***
## ---
## Signif. codes: 0 '***' 0.001 '**' 0.01 '*' 0.05 '.' 0.1 ' ' 1
##
## Residual standard error: 2.012 on 171 degrees of freedom
## (149 observations deleted due to missingness)
## Multiple R-squared: 0.06557, Adjusted R-squared: 0.04918
## F-statistic: 4 on 3 and 171 DF, p-value: 0.008761

etasq(father.absence.sex.Aus.income)

## Partial eta^2
## fath_ab 0.001972606
## income 0.063416764
## Residuals NA

“…and the significant interaction term for country… indicates that father absence before age 12 affects first coitus differently in these two samples. “

father.absence.sex.f.income <- lm(sex_yr ~ fath_ab * country +
 income, data = masc[masc$gender == "female", ])
summary(father.absence.sex.f.income)

##
## Call:
## lm(formula = sex_yr ~ fath_ab * country + income, data = masc[masc$gender ==
## "female", ])
##
## Residuals:
## Min 1Q Median 3Q Max
## -8.182 -1.182 -0.168 1.141 13.862
##
## Coefficients:
## Estimate Std. Error t value Pr(>|t|)
## (Intercept) 16.0227 0.4642 34.517 <2e-16 ***
## fath_ab0.5 0.1499 0.5090 0.294 0.7686
## fath_ab1 0.2051 0.4559 0.450 0.6532
## countryUnited State 0.3230 0.3245 0.995 0.3203
## income 0.2789 0.1699 1.641 0.1017
## fath_ab0.5:countryUnited State -0.4430 0.7739 -0.572 0.5675
## fath_ab1:countryUnited State -1.8636 0.6007 -3.102 0.0021 **
## ---
## Signif. codes: 0 '***' 0.001 '**' 0.01 '*' 0.05 '.' 0.1 ' ' 1
##
## Residual standard error: 2.11 on 302 degrees of freedom
## (183 observations deleted due to missingness)
## Multiple R-squared: 0.0657, Adjusted R-squared: 0.04713
## F-statistic: 3.539 on 6 and 302 DF, p-value: 0.002114

etasq(father.absence.sex.f.income)

## Partial eta^2
## fath_ab 0.027438632
## country 0.002168951
## income 0.008843042
## fath_ab:country 0.030964522
## Residuals NA

“There was no evidence of an effect of father absence on age at menarche in women… nor an interaction between country and father absence.”

father.absence.menarche.income <- lm(periodyr ~ fath_ab * country +
 income, data = masc)
summary(father.absence.menarche.income)

##
## Call:
## lm(formula = periodyr ~ fath_ab * country + income, data = masc)
##
## Residuals:
## Min 1Q Median 3Q Max
## -3.7612 -0.7720 0.2171 0.7956 4.8065
##
## Coefficients:
## Estimate Std. Error t value Pr(>|t|)
## (Intercept) 12.73946 0.24216 52.607 <2e-16 ***
## fath_ab0.5 -0.36625 0.27835 -1.316 0.1889
## fath_ab1 0.10225 0.24948 0.410 0.6821
## countryUnited State -0.32706 0.18366 -1.781 0.0756 .
## income 0.01086 0.09033 0.120 0.9043
## fath_ab0.5:countryUnited State 0.66154 0.46060 1.436 0.1516
## fath_ab1:countryUnited State -0.35369 0.34746 -1.018 0.3092
## ---
## Signif. codes: 0 '***' 0.001 '**' 0.01 '*' 0.05 '.' 0.1 ' ' 1
##
## Residual standard error: 1.439 on 469 degrees of freedom
## (102 observations deleted due to missingness)
## Multiple R-squared: 0.02354, Adjusted R-squared: 0.01105
## F-statistic: 1.885 on 6 and 469 DF, p-value: 0.0818

etasq(father.absence.menarche.income)

## Partial eta^2
## fath_ab 1.151857e-03
## country 1.106350e-02
## income 3.082356e-05
## fath_ab:country 8.254000e-03
## Residuals NA

“Regression revealed no significant associations between family ratings and age of first coitus in either sex…”

(men)

quality.fc.m.income <- lm(sex_yr ~ FRQ + income, data = masc_ageset[masc_ageset$gender ==
 "male", ])
summary(quality.fc.m.income)

##
## Call:
## lm(formula = sex_yr ~ FRQ + income, data = masc_ageset[masc_ageset$gender ==
## "male", ])
##
## Residuals:
## Min 1Q Median 3Q Max
## -4.9193 -1.4892 0.1718 1.4159 7.3272
##
## Coefficients:
## Estimate Std. Error t value Pr(>|t|)
## (Intercept) 17.3540 0.8477 20.472 <2e-16 ***
## FRQ 0.2195 0.2627 0.836 0.406
## income -0.2582 0.3148 -0.820 0.415
## ---
## Signif. codes: 0 '***' 0.001 '**' 0.01 '*' 0.05 '.' 0.1 ' ' 1
##
## Residual standard error: 2.309 on 78 degrees of freedom
## (52 observations deleted due to missingness)
## Multiple R-squared: 0.01665, Adjusted R-squared: -0.008566
## F-statistic: 0.6603 on 2 and 78 DF, p-value: 0.5196

etasq(quality.fc.m.income)

## Partial eta^2
## FRQ 0.008871741
## income 0.008553986
## Residuals

(women – but note interaction term)

quality.fc.f.income <- lm(sex_yr ~ FRQ * country + income, data = masc_ageset[masc_ageset$gender ==
 "female", ])
summary(quality.fc.f.income)

##
## Call:
## lm(formula = sex_yr ~ FRQ * country + income, data = masc_ageset[masc_ageset$gender ==
## "female", ])
##
## Residuals:
## Min 1Q Median 3Q Max
## -8.3041 -1.2810 -0.0876 1.1145 13.8280
##
## Coefficients:
## Estimate Std. Error t value Pr(>|t|)
## (Intercept) 15.95404 0.43625 36.571 < 2e-16 ***
## FRQ 0.01556 0.16791 0.093 0.92620
## countryUnited State -0.17624 0.23506 -0.750 0.45392
## income 0.30934 0.16119 1.919 0.05583 .
## FRQ:countryUnited State 0.59388 0.22477 2.642 0.00863 **
## ---
## Signif. codes: 0 '***' 0.001 '**' 0.01 '*' 0.05 '.' 0.1 ' ' 1
##
## Residual standard error: 2.089 on 333 degrees of freedom
## (220 observations deleted due to missingness)
## Multiple R-squared: 0.04983, Adjusted R-squared: 0.03841
## F-statistic: 4.366 on 4 and 333 DF, p-value: 0.001873

etasq(quality.fc.f.income)

## Partial eta^2
## FRQ 0.023236034
## country 0.003038133
## income 0.010938578
## FRQ:country 0.020533080
## Residuals NA

“… or menarche in women”

quality.menarche.income <- lm(periodyr ~ FRQ * country + income,
 data = masc_ageset)
summary(quality.menarche.income)

##
## Call:
## lm(formula = periodyr ~ FRQ * country + income, data = masc_ageset)
##
## Residuals:
## Min 1Q Median 3Q Max
## -3.7193 -0.7627 0.1316 0.7003 4.0750
##
## Coefficients:
## Estimate Std. Error t value Pr(>|t|)
## (Intercept) 12.88624 0.23589 54.628 <2e-16 ***
## FRQ -0.12106 0.08710 -1.390 0.1652
## countryUnited State -0.27332 0.13284 -2.057 0.0402 *
## income -0.05158 0.08761 -0.589 0.5563
## FRQ:countryUnited State 0.22011 0.12596 1.747 0.0812 .
## ---
## Signif. codes: 0 '***' 0.001 '**' 0.01 '*' 0.05 '.' 0.1 ' ' 1
##
## Residual standard error: 1.406 on 499 degrees of freedom
## (187 observations deleted due to missingness)
## Multiple R-squared: 0.01716, Adjusted R-squared: 0.009281
## F-statistic: 2.178 on 4 and 499 DF, p-value: 0.07035

etasq(quality.menarche.income)

## Partial eta^2
## FRQ 0.0002215059
## country 0.0096625979
## income 0.0006941751
## FRQ:country 0.0060823348
## Residuals NA

“…except for an interaction term between family relationship scores and country in predicting age of first coitus in women: positive ratings of family relationships were associated with a higher age of first coitus in American women…”

# US women
quality.sex.US.income <- lm(sex_yr ~ FRQ + income, data = masc_ageset[(masc_ageset$gender ==
 "female") & (masc_ageset$country == "United State"), ])
summary(quality.sex.US.income)

##
## Call:
## lm(formula = sex_yr ~ FRQ + income, data = masc_ageset[(masc_ageset$gender ==
## "female") & (masc_ageset$country == "United State"), ])
##
## Residuals:
## Min 1Q Median 3Q Max
## -8.0514 -1.4936 -0.1337 1.3890 6.1825
##
## Coefficients:
## Estimate Std. Error t value Pr(>|t|)
## (Intercept) 16.8843 0.6350 26.588 <2e-16 ***
## FRQ 0.4931 0.1672 2.949 0.0037 **
## income -0.1057 0.2285 -0.462 0.6444
## ---
## Signif. codes: 0 '***' 0.001 '**' 0.01 '*' 0.05 '.' 0.1 ' ' 1
##
## Residual standard error: 2.132 on 151 degrees of freedom
## (52 observations deleted due to missingness)
## Multiple R-squared: 0.07172, Adjusted R-squared: 0.05942
## F-statistic: 5.833 on 2 and 151 DF, p-value: 0.00363

“…but not Australian women”

# Aus women
quality.sex.Aus.income <- lm(sex_yr ~ FRQ + income, data = masc_ageset[(masc_ageset$gender ==
 "female") & (masc_ageset$country == "Australia"), ])
summary(quality.sex.Aus.income)

##
## Call:
## lm(formula = sex_yr ~ FRQ + income, data = masc_ageset[(masc_ageset$gender ==
## "female") & (masc_ageset$country == "Australia"), ])
##
## Residuals:
## Min 1Q Median 3Q Max
## -3.4269 -1.2004 -0.1983 0.8801 13.2705
##
## Coefficients:
## Estimate Std. Error t value Pr(>|t|)
## (Intercept) 14.82212 0.58666 25.265 < 2e-16 ***
## FRQ 0.09566 0.16457 0.581 0.561803
## income 0.75661 0.22420 3.375 0.000904 ***
## ---
## Signif. codes: 0 '***' 0.001 '**' 0.01 '*' 0.05 '.' 0.1 ' ' 1
##
## Residual standard error: 2.016 on 181 degrees of freedom
## (168 observations deleted due to missingness)
## Multiple R-squared: 0.0595, Adjusted R-squared: 0.04911
## F-statistic: 5.726 on 2 and 181 DF, p-value: 0.00388
